# *Elateriospermum tapos* Yoghurt as a Therapeutic Intervention for Obesity-Associated Cognitive Impairments and Anxiety-like Behaviour in a High Fat Diet Maternal Obese Rat Model

**DOI:** 10.3390/nu15102312

**Published:** 2023-05-15

**Authors:** Ruth Naomi, Soo Huat Teoh, Rusydatul Nabila Mahmad Rusli, Hashim Embong, Hasnah Bahari, Jaya Kumar

**Affiliations:** 1 Department of Human Anatomy, Faculty of Medicine and Health Sciences, Universiti Putra Malaysia, Serdang 43400, Malaysia; gs60018@student.upm.edu.my (R.N.); rusydatulnabila17@gmail.com (R.N.M.R.); 2 Advanced Medical and Dental Institute, Universiti Sains Malaysia, Penang 13200, Malaysia; soohuat@usm.my; 3 Department of Emergency Medicine, Faculty of Medicine, Universiti Kebangsaan Malaysia, Kuala Lumpur 56000, Malaysia; hashimembong77@ukm.edu.my; 4 Department of Physiology, Faculty of Medicine, Universiti Kebangsaan Malaysia, Kuala Lumpur 56000, Malaysia

**Keywords:** yoghurt, nutritional intervention, high-fat diet, maternal obesity, anxiety, cognition

## Abstract

Maternal obesity can be considered an intergenerational cycle and is also an important indicator of cognitive impairments. It is thought that using natural products is the best and safest way to combat maternal obesity and associated complications. Recent studies have shown that *Elateriospermum tapos* (*E. tapos*) contains bioactive compounds with anti-obesity effects, and yoghurt is a convenient medium for supplementing obese maternal rats with *E. tapos* extract. Thus, the aim of this study is to investigate the impact of *E. tapos* in yoghurt on maternally obese rats’ cognitive function supplemented with a high-fat diet (HFD). In this study, 48 female Sprague-Dawley rats were used. The rats were fed HFD for a period of 16 weeks to induce obesity, after which they were allowed to mate. Upon confirmation of pregnancy, obese rats were given varying doses of *E. tapos* (5, 50, and 500 mg/kg) in yoghurt until postnatal (PND) day 21. On PND 21, the dams’ body mass index (BMI), Lee index, abdominal circumference, oxidative status, and metabolic profile were measured. The behavioral tests (open field, place, and object recognition) were conducted on PND 21 to access memory. The results show that the 50 and 500 mg/kg *E. tapos* in yoghurt supplemented groups had similar BMI, Lee index, abdominal circumference, lipid profile, FBG, insulin, FRAP, and GSH levels, as well as a similar recognition index, in comparison with the control group supplemented with saline. In conclusion, the results of this study indicate that the newly formulated *E. tapos* in yogurt can act as an anti-obesity agent in maternal obesity, alleviate anxiety, and enhance hippocampal-dependent memory.

## 1. Introduction

Obesity has become a widespread problem worldwide, particularly in children. Global statistics on obesity prevalence indicate that around 42 million children were classified as obese in 2013, while the World Health Organization (WHO) reported that 600 million adults were obese. The WHO defines obesity as having a body mass index (BMI) of 30 or higher [1]. Childhood obesity arises from maternal obesity. This is because maternal obesity, also termed metainflammation [2], is an intergenerational vicious cycle [3]. The high intake of high-fat diets (HFD) may be a contributing factor in the high body weight retention experienced during pregnancy. Consuming HFD when pregnant has been linked to increased adiposity, inflammatory changes in multiple important organs, and an increase in the synthesis of hepatic glucose [4]. In HFD-induced maternal obesity, insulin resistance, diminished glucose tolerance, and alterations in oxidative stress markers are some of the typical pathogenic abnormalities reported [5]. Such alterations in obese patients may manifest as diminished cognition [6] and elevated anxiety levels [7]. According to studies, BMI has a direct influence on both cognitive and physical abilities. This is mostly because obesity causes morphological and functional alterations in the brain. In the presence of a high concentration of visceral fats, the volume of the hippocampus will decrease while the ventricular volume tends to increase [8]. Meanwhile, central adiposity has proven to have a positive association with cognitive decline [9], and BMI is one of the indirect measures of central obesity [8]. This is because central adiposity compromises the neural integrity and deteriorates the structure of the hippocampus, thalamus, and midbrain. Along with this, the blood flow to the prefrontal cortex declines over time with increasing BMI. Hence, the functionality related to episodic memory will eventually be impaired, manifesting as poor cognitive performance [10]. Thus, it is speculated that curbing maternal obesity could alleviate memory decline in HFD-induced obese dams.

The current modern approach for treating obesity, such as orlistat and sibutramine drugs, is said to be less effective. This is because studies show that individuals tend to regain weight upon stopping taking orlistat, and severe diarrhoea accompanied by abdominal cramps is one of the cases referred in a hospital setting [11]. Meanwhile, sibutramine is associated with severe adverse effects such as asthenia, obstipation, insomnia, dry mouth, and mood swings due to the interference of sibutramine in brain circuits [12]. Due to the sparse efficacy of modern medicines, a natural product will be the best option. One reason is that natural treatments are often perceived as being safer and more gentle on the body, as they are derived from natural sources and have typically been used for centuries. Synthetic drugs, on the other hand, are often associated with side effects and adverse reactions due to their chemical composition. In these cases, the medicinal plant is considered to be one of the safest and best options to curb maternal obesity and its complications due to the safety and efficacy of medicinal plant extract in maternal parents [13]. At the same time, the presence of live bacterial cultures in yoghurt is proven to reverse memory decline and improve brain function in obese individuals [14]. As such, yoghurt could be the most effective medium to deliver bioactive compounds in natural extracts. A preliminary study shows that *Elateriospermum tapos* (*E. tapos*), which is commonly found in Southeast Asia, contains a high concentration of antioxidants known as flavonoids [15] and some other bioactive molecules that can prevent the accumulation of fats by suppressing the activity of lipoprotein lipase [16]. Alkaloids, tannins, saponins, sterols, iodine, and linolenic acids are a few additional isolated bioactive molecules from *E. tapos* extract [17]. After conducting a literature review, we realized that the optimal dietary intervention to prevent memory loss in HFD-induced obesity in maternal rats would be to incorporate a local medicinal plant (*E. tapos*) into yoghurt that comprised live bacterial cultures. Therefore, the goal of this study is to show how *E. tapos* in yoghurt can aid dams with HFD-induced obesity by mitigating their cognitive deficits.

## 2. Materials and Methods

### 2.1. E.tapos Seed Extraction Using Ethanol

The seeds of *E. tapos* were obtained from the research center of the Forest Research Institute of Malaysia (FRIM). To ensure their purity, the seeds were then sent to the Herbarium Biodiversity Unit at UPM and assigned the voucher code UPM SK 3154/17. Following confirmation of purity, the seeds were washed with tap water and air-dried at room temperature. Approximately 500 g of *E. tapos* seeds were then immersed in 2 L of 95% ethanol for 7 days. After 7 days, the resulting filtrate was collected, filtered using Whatman paper, and subjected to extraction with a rotary evaporator under reduced pressure [18]. This procedure was repeated three times, and the resulting crude extract was gathered, consolidated, and blended with maltodextrin powder at a ratio of 1:1. The mixture was then desiccated overnight. The *E. tapos* powder was collected the following day and stored at −20 °C.

### 2.2. E. tapos Yoghurt Preparation

The yoghurt formulation was optimized from a previous study conducted by Naomi et al. in 2023 [19]. To formulate *E. tapos* yoghurt, 100 mL of full-cream milk obtained from Dutch Lady Pure-farm UHT was heated to 75 °C and boiled for 30 min. The mixture was then cooled to room temperature until it reached 45 °C. Next, a starter culture consisting of live bacterial strains was added to the boiled milk, and the mixture was placed in a yoghurt maker (Pensonic PYM-700) and incubated for 7 to 8 h. The resulting yoghurt was then chilled in the refrigerator overnight at a temperature of 4 °C. The next day, *E. tapos* powder was added [20] and incorporated into the yoghurt at a ratio of 2 g per 100 mL [21].

### 2.3. TWIMS-QTOFMS Analysis for E. tapos Yoghurt

The UHPLC system was coupled to a Vion IMS QTOF hybrid mass spectrometer from Waters, equipped with a Lock Spray ion source. The ion source was operated in negative electrospray ionization (ESI) mode under the following specific conditions: capillary voltage, 1.50 kV; reference capillary voltage, 3.00 kV; source temperature, 120 °C; desolvation gas temperature, 550 °C; desolvation gas flow, 800 L/h; and cone gas flow, 50 L/h. Nitrogen (>99.5%) was employed as desolvation and cone gas. The data were acquired in high-definition MS^E^ (HDMS^E^) mode in the range *m*/*z* 50–1500 at 0.1 s/scan. Thus, two independent scans with different collision energies (CE) were alternatively acquired during the run: a low-energy (LE) scan at a fixed CE of 4 eV and a high-energy (HE) scan where the CE was ramped from 10 to 40 eV. Argon (99.999%) was used as a collision-induced dissociation (CID) gas [22].

### 2.4. Preparation of HFD

The HFD was produced by, as described elsewhere [23], blending 6% corn oil, 6% ghee, 20% milk powder, and 68% standard rat pellets. The mixture was subsequently baked at 60 °C for 1 to 2 h and refrigerated overnight [23].

### 2.5. Experimental Animals

In compliance with the UPM/IACUC/AUP-R025/2022 code of conduct, all animal procedures were conducted only with the approval of the Institutional Animal Care and Use Committee (IACUC) at Universiti Putra Malaysia. Young female Sprague Dawley (SD) rats that were 6 weeks old and weighed between 150 and 200 g were used in this investigation (n = 48). All rats underwent a one-week acclimatization period in controlled light and dark cycles (12 h each), at room temperature, with a humidity of 22 ± 3 °C. Throughout the acclimatization period, during which the rats had unrestricted access to water provided through bottle feeding, all rats were nourished using normal rat pellets (Gold Coin Feedmills (M)) [24].

### 2.6. Obesity Induction

This study involved feeding 40 female SD rats with pre-prepared HFD pellets for a period of 16 weeks to induce obesity. Meanwhile, the control group received standard rat chow pellets and unrestricted access to water through bottle feeding. The marked elevation in BMI observed in the HFD-fed group, when compared to the group supplemented with standard rat chow, confirms the successful induction of obesity. The HFD groups were supplemented with HFD up to PND 21.

### 2.7. Mating

Following successful induction of obesity, the female rats were placed in the same cage with male SD rats (2:1) to allow mating. The subsequent morning, all female rats underwent manual abdominal palpation and vaginal smears to determine pregnancy. Vaginal smears were examined under a microscope at a magnification of ×100 (KF2; Carl Zeiss, Hamburg, Germany). The first day on which sperm were detected was recorded as day 0 post coitum [25].

### 2.8. Gestation, Weaning, and Treatment Groups

Upon confirmation of pregnancy, the rats were divided into different treatment groups and administered the corresponding treatment until postnatal day (PND) 21. The treatment groups consisted of: normal chow and saline (NS), HFD and saline (HS), HFD and plain yoghurt (HY), HFD and 5 mg/kg of *E. tapos* in yoghurt (HYT5), HFD and 50 mg/kg of *E. tapos* in yoghurt (HYT50), and HFD and 500 mg/kg of *E. tapos* in yoghurt (HYT500) [19]. The yoghurt was administered once daily at 8 a.m. via oral gavage during gestation until PND 21. The volume of yoghurt given was equivalent to 1% of the rat’s total body weight, with the concentration of the yoghurt being maintained according to the specified group.

### 2.9. Morphometric Measurements

The abdominal circumference measured from the anterior to the forefoot, the thoracic circumference, and the length of the SD rats were measured from the nose to the anus on PND 21. All lengths were recorded in centimeters. The recorded measurements were used to calculate BMI and Lee’s index. The formula for BMI is calculated by dividing body weight in grams by the square of the length in centimeters. A BMI greater than 0.687 g/cm^2^ is considered obese [26]. The formula for the Lee index is calculated by taking the cube root of the body weight in grams divided by the nose-to-anus length in centimeters. A Lee index value greater than 310 g is considered obese [27].

### 2.10. Anxiety Test

On PND 21, anxiety tests were performed on the rats. A gray PVC open box, measuring 80 cm in width, 80 cm in length, and 50 cm in height, was used to house the rats. The test was conducted only once during the light phase of a homogenous illumination cycle with a 100-lux intensity. The rats were placed in a specific corner of the box and allowed to explore for 5 min. The ANY-maze™ Video Tracking System (Stoelting Co., Wood Dale, IL, USA) was used to record various parameters during the experiment, including total distance traveled, time spent at the center, and peripheral zone [28].

### 2.11. Novel Object and Place Recognition Test

On PND 23, all dams were subjected to the novel object recognition test (NORT) following a previously described protocol [29]. During the NORT experiment, two different objects, 1.25-L plastic bottles and porcelain mugs, were used. The rats were given 10 min to acclimate to the open field box for the first two days before the test. On the third day, the rats were given 5 min to explore two identical objects, followed by a retention phase of another 5 min. The objects were then placed in the same spot for the NORT test, with one object being familiar and the other novel. In the PRT test, both objects were identical, but one was placed in a different location. The ANY-maze™ Video Tracking System (Stoelting Co., Wood Dale, IL, USA) was used to record the time spent exploring novel objects and new places for a duration of 5 min during both tests. The exploration ratio is calculated as the time spent at a novel object or place divided by the sum of time spent at both the novel and familiar objects or places. If the exploration ratio is greater than 0.50, it is considered a novelty preference [29].

### 2.12. Fasting Blood Glucose Level

On postnatal day 28 (PND 28), the rats were subjected to a 12-h fast but were provided with free access to water. The next day, blood samples were collected by pricking their tails. Blood was collected into glucose strips, and a glucometer (Glucocard™ 01-mini) was used to measure the glucose level. The readings were recorded [30].

### 2.13. Insulin Level

The rats were euthanized through a carbon dioxide overdose, and their hypothalamus was promptly collected and snap-frozen for preservation. Heparin tubes were used to collect blood samples, which were then centrifuged at 3500 rpm for 15 min. The resulting plasma was then transferred to a plain tube. Commercial rat insulin ELISA kits, provided by Shibayagi Co., Ltd., Gunma, Japan, were utilized to measure the insulin levels in the plasma.

### 2.14. Lipid Profile

The levels of triglycerides, total cholesterol, LDL, and HDL were analyzed using a diagnostic reagent test kit obtained from Roche, Germany, with a Hitachi Automatic Analyzer 902 (Tokyo, Japan) [31].

### 2.15. Oxidative Stress Markers

The oxidative status was measured in serum and hypothalamus as described in a previous study by Naomi et al., 2023 [32]. To determine the hypothalamic levels of FRAP and GSH, the hypothalamus was minced and diluted with ice-cold phosphate buffer saline (0.02 M) with a pH of 7 at a ratio of 1:15 (*w*/*v*). The samples were then homogenized on ice using a glass homogenizer (Omni TH, Omni International, Kennesaw, GA, USA) and sonicated three times using an ultrasonic cell disrupter (UP 400S) for 20 s each time [19]. The supernatants collected after centrifugation were analyzed for FRAP and GSH in both hypothalamus samples and serum using ELISA kits from Cayman Chemical Company [33].

### 2.16. Statistical Analysis

SPSS version 27.0 was utilized for statistical analysis, and results were presented as mean ± standard error of the mean (SEM). Prior to conducting a one-way ANOVA, normality tests were performed. Tukey’s post hoc test was utilized to assess the significance of group differences. Results with probabilities of *p* < 0.05 were considered statistically significant.

## 3. Results

### 3.1. Analysis of Bioactive Compounds of E. tapos Yoghurt

The chromatograms of bioactive compound quantification from one mL of *E. tapos* yoghurt and their peak maxima are attached in the Supplementary File. Table 1 shows the isolated bioactive compounds from *E. tapos* yoghurt. Approximately 20 bioactive compounds have been identified in *E. tapos* yoghurt and found to have a profound effect on fat tissue and cognition. Table 1. Bioactive compounds of *E. tapos* yoghurt.

### 3.2. BMI, Abdominal Circumference, and Lee Index Obese Dams

**Figure 1:**
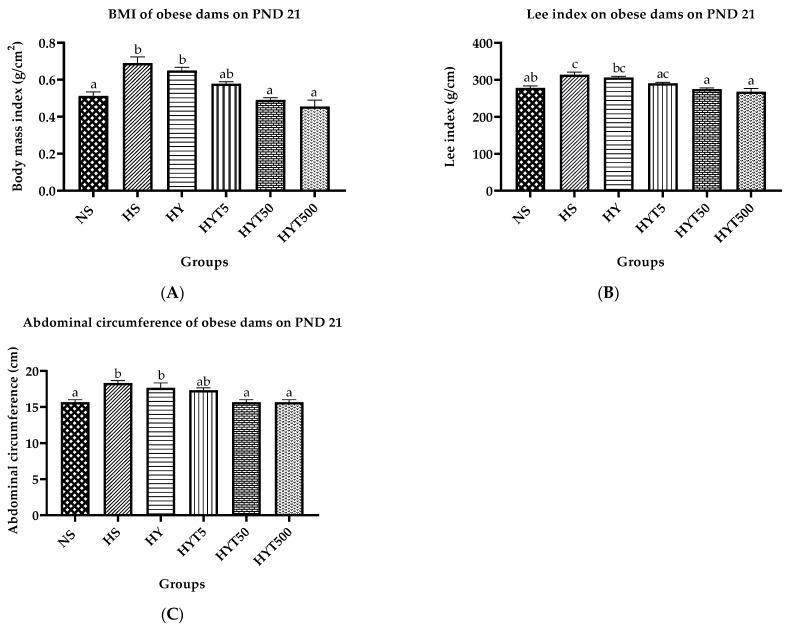
(**A**) BMI of obese dams. (**B**) Lee index of obese dams. (**C**). Abdominal circumference of obese dams. Different letters among groups indicate significant differences (*p* < 0.05).

Figure 1A–C display the results of the study regarding the BMI, Lee index, and abdominal circumference of obese dams treated with different concentrations of *E. tapos* in yoghurt. The findings suggest that the BMI, Lee index, and abdominal circumference of the HS and HY groups are significantly higher than those of the NS. On the other hand, the BMI, abdominal circumference, and Lee index of HYT50 and HYT500 are significantly lower than those of HS and HY. Furthermore, there are no significant differences in the BMI, abdominal circumference, and Lee index of HYT5, HY50, and HYT500 compared to NS.

### 3.3. Anxiety Test in Obese Dams on PND 21

**Figure 2:**
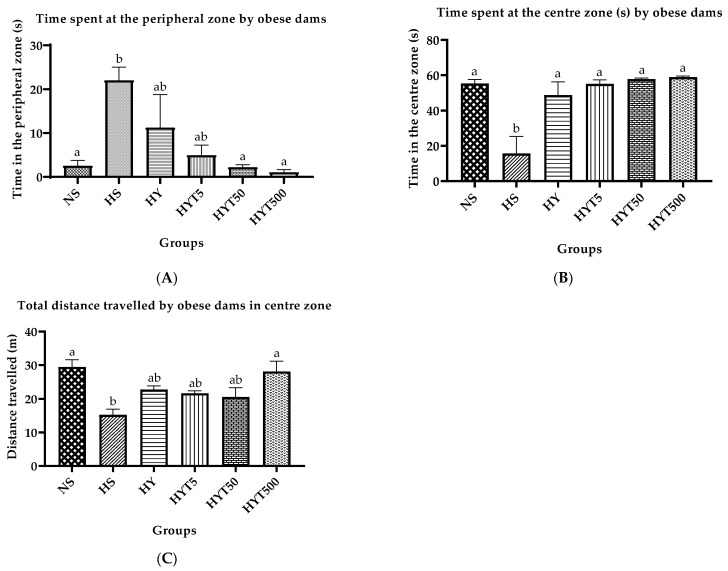
(**A**) Time spent by obese dams at the peripheral zone in an OFT. (**B**) Time spent by obese dams at the center zone in an OFT. (**C**) Total distance traveled by obese dams in an OFT. Different letters among groups indicate significant differences (*p* < 0.05).

Figure 2A–C depict the time spent by obese dams in the peripheral zone, center zone, and the total distance traveled during the OFT. The results show that obese dams in the HS group spent significantly more time (*p* < 0.05) in the peripheral zone compared to dams in the NS group, while dams in the HYT50 and HYT500 groups spent significantly less time (*p* < 0.05) in the peripheral zone compared to HS. There was no significant difference (*p* > 0.05) in the time spent in the peripheral zone between the HY, HYT5, HY50, and HYT500 groups compared to NS. Similarly, obese dams in the HS group spent significantly less time (*p* < 0.05) in the center zone compared to dams in the NS. Dams in the HY, HYT5, HYT50, and HYT500 spent significantly less time (*p* < 0.05) in the peripheral zone compared to the HS. The mean time spent in the center zone by dams in the HY, HYT5, HYT50, and HYT500 showed no significant difference compared to dams in the NS. Finally, the total distance traveled by obese dams in the HS group during OFT was significantly lower (*p* < 0.05) compared to dams in the NS group. There was no significant difference (*p* > 0.05) in the total distance traveled by dams in the HY, HYT5, and HYT50 compared to dams in the HS and NS. The mean total distance traveled by dams in the HYT500 group was similar to dams in the NS.

### 3.4. Novel Object, and Place Recognition Test (PRT) in Obese Dams

**Figure 3:**
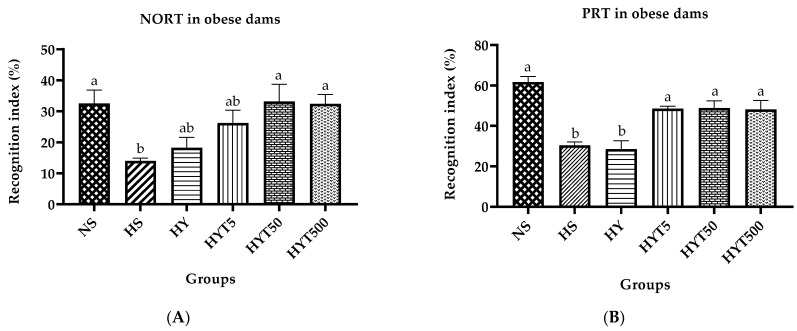
(**A**) The recognition index of obese dams in NORT. (**B**) The recognition index of obese dams in PRT. Different letters among groups indicate significant differences (*p* < 0.05).

Figure 3A,B present the recognition index (%) of obese dams in the NORT and the PRT, respectively. Figure 3A shows that obese dams in HS spent significantly less time recognizing the novel object in NORT compared to dams in NS (*p* < 0.05). There was no significant difference (*p* > 0.05) in the recognition index of dams in HY and HYT5 compared to HS and NS. The mean value for the recognition index of dams in HYT50 and HYT500 in NORT was similar to that of the NS. In Figure 3B, the recognition of obese dams in HY and HS in PRT was significantly lower (*p* < 0.05) compared to dams in the NS group. On the other hand, the recognition of HYT5, HYT50, and HYT500 was significantly higher (*p* < 0.05) compared to dams in HY and HS in PRT, with a similar mean value to dams in the NS.

### 3.5. Fasting Blood Glucose in Obese Dams

**Figure 4:**
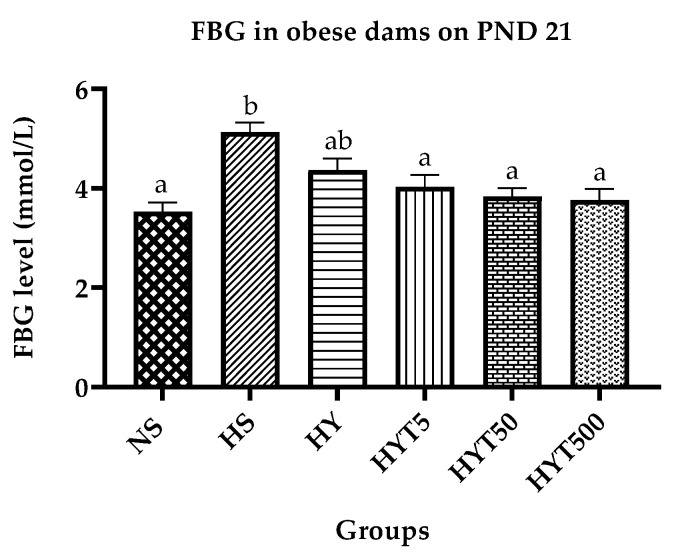
Fasting blood glucose level in obese dams. Different letters among groups indicate significant differences (*p* < 0.05).

In Figure 4, the fasting blood glucose (FBG) levels of obese dams are presented. It is shown that dams in the HS have significantly higher FBG (*p* < 0.05) compared to dams in the NS. The FBG levels of dams in the HY group show no significant difference (*p* > 0.05) compared to both the HS and NS. On the other hand, dams in the HYT5, HYT50, and HYT500 groups have significantly lower FBG levels (*p* < 0.05) compared to the HS. There is no significant difference (*p* > 0.05) in the FBG levels of dams in the HYT5, HY50, and HYT500 groups compared to the NS on PND 21.

### 3.6. Insulin Level in Obese Dams

**Figure 5:**
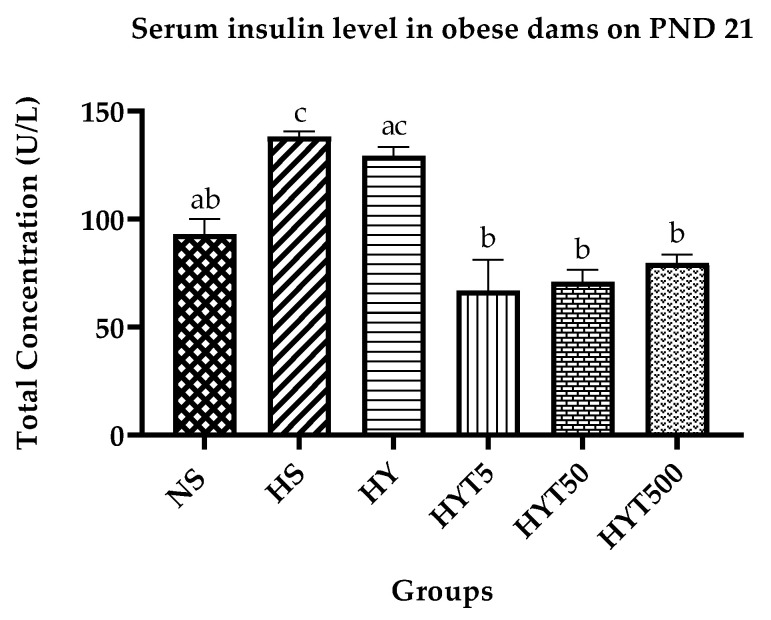
Insulin level in obese dams. Different letters among groups indicate significant differences (*p* < 0.05).

The results presented in Figure 5 demonstrate the serum insulin concentration in dams treated with various concentrations of *E. tapos* in yoghurt. The serum insulin level of obese dams in the HS is significantly higher (*p* < 0.05) than in the NS. The serum insulin concentration of dams in the HY group shows no significant difference (*p* > 0.05) compared to both the HS and NS. However, the serum insulin level of dams in HYT5, HYT50, and HYT500 is significantly lower (*p* < 0.05) than in the HS and HY. There is no significant difference (*p* > 0.05) in the serum insulin concentration of dams in HYT5, HY50, and HYT500 compared to the NS on PND 21.

### 3.7. Lipid Profile in Obese Dams on PND 21

**Figure 6:**
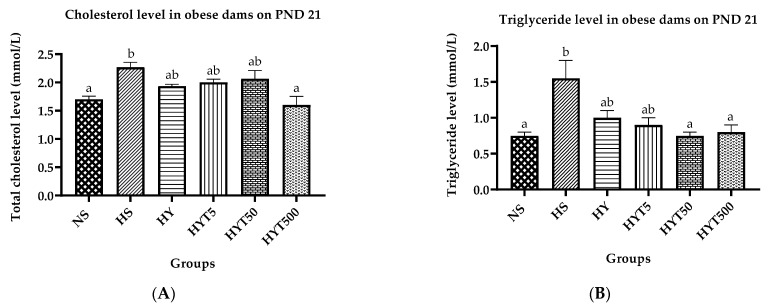

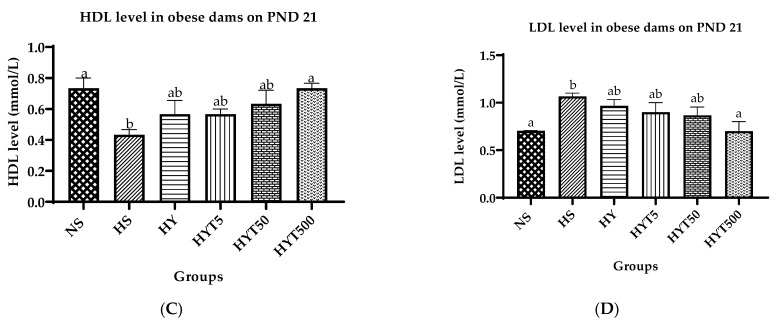
(**A**) Serum cholesterol level in obese dams. (**B**) Serum triglyceride level in obese dams. (**C**) Serum HDL level in obese dams. (**D**) Serum LDL level in obese dams. Different letters among groups indicate significant differences (*p* < 0.05).

Figure 6A–D present the lipid profile analysis of obese dams that received different concentrations of *E. tapos* in yoghurt. The results show that on PND 21, the serum cholesterol level of dams in the HS is significantly higher than the NS. Meanwhile, there is no significant difference in the serum cholesterol levels of dams in HY, HYT5, and HYT50 compared to both HS and NS. On the other hand, the serum cholesterol level of dams in the HYT500 is significantly lower than the HS; however, there is no significant difference compared to the NS. Regarding the serum triglyceride level, the HS showed a significantly higher level than the NS, while the HY and HYT5 did not show a significant difference compared to both the HS and the NS. The serum triglyceride level of dams in the HYT50 and HYT500 was significantly lower than the HS, with no significant difference compared to the NS. The serum HDL level of dams in the HS was significantly lower than the NS, and the HY, HYT5, and HYT50 did not show a significant difference compared to both the HS and the NS. In contrast, the serum HDL level of dams in the HYT500 was significantly higher than the HS; however, no significant difference was found compared to the NS. Finally, the serum LDL level of dams in the HS was significantly higher than the NS and the HY, while HYT5 and HYT50 did not show a significant difference compared to both the HS and the NS. The serum LDL level of dams in the HYT500 was significantly lower than the HS, with no significant difference compared to the NS on PND 21.

### 3.8. Oxidative Stress Markers in Serum and Hypothalamus

**Figure 7:**
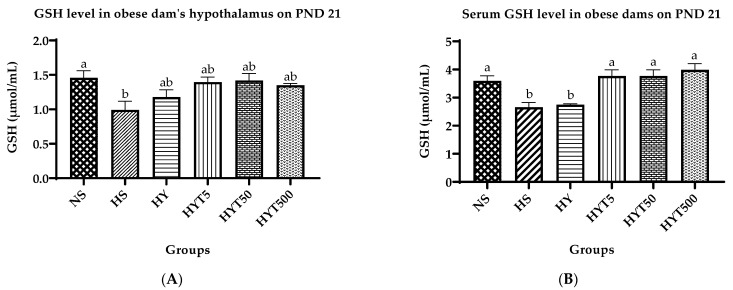

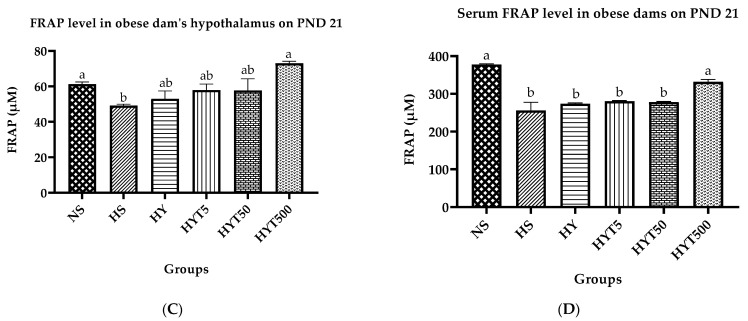
(**A**) GSH level in obese dam’s hypothalamus. (**B**) Serum GSH level in obese dams. (**C**) FRAP level in obese dam’s hypothalamus. (**D**) Serum GSH level in obese dams. Different letters among groups indicate significant differences (*p* < 0.05).

Figure 7A–D depict the alterations in oxidative stress markers in the serum and hypothalamus of dams given *E. tapos* in yoghurt. In Figure 7A, it is shown that the GSH level in the hypothalamus of dams in the HS is significantly lower (*p* < 0.05) than that in the NS. However, the GSH level in the hypothalamus of dams in the HY, HYT5, HYT50, and HYT500 does not differ significantly (*p* > 0.05) from that of the HS and NS on PND 21. Figure 7B shows that the serum GSH level of obese dams in the HY and HS is significantly lower (*p* < 0.05) than that of dams in the NS, while the serum GSH concentration in the HYT5, HYT50, and HYT500 is significantly higher (*p* < 0.05) than that of dams in the HY and HS and is comparable to that of dams in the NS. Similarly, in Figure 7C, the FRAP level in the hypothalamus of obese dams in the HS is significantly lower (*p* < 0.05) than that in the NS. However, the FRAP level in the hypothalamus of dams in the HY, HYT5, and HYT50 groups does not differ significantly (*p* > 0.05) from that of the HS and NS on PND 21. On the other hand, the FRAP level in the hypothalamus of dams in the HYT500 is significantly higher (*p* < 0.05) than that in the HS and is similar in mean value to that in the NS. Finally, Figure 7D demonstrates that the serum FRAP level in obese dams in the HY, HS, HYT5, and HYT50 is significantly lower (*p* < 0.05) than that in the NS. Meanwhile, the serum FRAP concentration in the HYT500 is significantly higher (*p* < 0.05) than that in the HY, HS, HYT5, and HYT50 and is comparable in mean value to that in the NS.

## 4. Discussion

One of the prime contributors to memory loss is obesity, and having a high BMI raises your likelihood of developing dementia or Alzheimer’s in later life. This is since a high degree of adiposity in obesity may directly influence brain regions involved in memory, such as the hippocampus and frontotemporal region, leading to memory deficits [34]. Since the obese gene is transferred from one generation to the next through epigenetic alterations, obesity can be categorized as a transgenerational cycle [35]. Consequently, the best option for reducing obesity and its complications may be to prevent the transmission of the obese gene from maternal parents. Thus, the management of obesity should focus on pregnancy itself because pre-pregnancy weight gain tends to persist even after weaning [36]. Consumption of fat-free yoghurt has been proven to enhance fat loss by up to 22% [37], while medicinal plant extracts such as *E. tapos* have been proven to improve cognitive performance [38]. As such, in this study, we integrated medicinal plant extract into yoghurt containing live bacteria and investigated the outcome of *E. tapos* in yoghurt on maternal obesity-induced cognitive decline in obese dams using rodents. Toxicological evaluation of *E. tapos* yoghurt shows no toxic effect up to 2000 mg/kg consumption [17]. In the first phase of the study, the significant difference in BMI in the HS group confirmed the weight retention after delivery, and the BMI above 0.68 g/cm^2^ in HS proves the dams were obese [26]. To boost this, the Lee index of dams in the HS group >310 g with a significantly high abdominal circumference and dysregulated lipid profile further supports the theory of weight retention/obesity on PND 21 [27], thereby proving the successful establishment of the obese dams model in the first phase of this study.

The *E. tapos* yoghurt supplement during the gestational duration in the HYT50 and HYT500 in this study was successful in preventing the retention of body weight with a similar BMI, Lee index, and abdominal circumference as in the dams of the NS group. This is due to the abundance of various types of flavonoid compounds in the ethanol-extracted *E. tapos* seed, including *ginkgetin*, *kaempferol*, *amentoflavone*, *putraflavone*, and *sequoiaflavone* [39], which supports the inhibitory activity of pancreatic lipase, -glucosidase, and -amylase. This may help the body’s weight loss system by preventing the absorption of fats and carbs in the gastrointestinal tract [15]. The perfect BMI, Lee index, and abdominal circumference in the dams of the HYT50 and HYT500 on PND 21 may have this as their underlying reason. In addition, *E. tapos* seed extract contains the highest concentration of protein (16.10%) in comparison with most medicinal plant extracts [40], which enhances the feeling of fullness and satiety to a greater extent, thereby preventing excessive calorie intake [41]. *E. tapos* seed extract is composed of a high amount (29.83%) of unsaturated fatty acids [42], which aid in the removal of bad cholesterol (LDL) by the liver. In such conditions, the liver tends to prioritize the transformation of polyunsaturated fatty acids into ketone bodies over LDL. As a result, cholesterol and LDL levels tend to be lower in the serum [43]. The presence of an appreciable amount of omega-3 essential fatty acid in *E. tapos* [40] further enhances the process of fatty acid oxidation by reducing the triglyceride content in the serum and suppressing hepatic lipogenesis [44]. Concurrently, omega-3 essential fatty acids could stimulate the activity of lipoprotein lipase and expedite the hydrolysis of triglycerides. This may eventually increase the serum HDL concentration [45]. This evidence is observed in this study since the highest concentration of *E. tapos* in the yoghurt supplemented dams group (HYT50 and HYT500) shows similar serum LDL, triglycerides, and cholesterol values to the dams in the NS groups, which are significantly lower compared to HS and HY. The serum HDL concentration in dams of HYT50 and HYT500 was restored with a similar mean value to the dams in the NS. However, a preliminary study on *E. tapos* yoghurt found that it contains 1.6 × 10^5^ CFU/g of *Lactobacillus species* and has a titratable acidity of 0.432 CFU/g of lactic acid. This suggests that *E. tapos* yoghurt can stimulate *Lactobacillus* growth in the intestine, which can improve immune function and aid in weight loss by balancing gut microbes [17].

In addition, the increased number of adipocytes in obese individuals will release an excessive amount of free fatty acids into the bloodstream. High levels of free fatty acids will encourage insulin production, which is mediated by glucose. Free fatty acids also impede the inhibition of insulin-modulated glycogenolysis, which results in insulin resistance [46]. The performance of the cell may be hampered by insulin resistance [47]. This may indicate a rise in insulin and FBG levels in the blood, as shown in the dams of the HS, thus supporting the construction of the maternal obesity model as successful in this investigation. However, the oral administration of *E. tapos* in yoghurt in HYT5, HYT50, and HYT500 during the gestational period in obese dams shows a similar mean value of serum FBG and insulin compared to dams in the NS. The possible reason for this could be the existence of unsaturated fatty acids, such as linolenic acid and oleic acid, in the *E. tapos* seed [40]. It has been proven that linolenic acids can gradually normalize glucose levels and insulin tolerance within 5 weeks by inducing changes in the mitochondrial subsarcolemma and enhancing gene transcription involved in insulin sensitivity [48]. Similarly, oleic acids in *E. tapos* yoghurt impede glucose production as well as inhibit the expression of neuropeptide Y in the hypothalamus, thereby delaying satiety [49].

In contrast, the results of the anxiety test in this study demonstrate that obese dams who had been given an HFD (HS group) exhibited thigmotactic behavior, spending much more time in the peripheral zone and avoiding the center of the open field. Elevated time spent in the peripheral zone in fat dams is a sign of high anxiety levels [50]. According to studies, maternal obesity is strongly correlated with the severity of anxiety and alterations in mood [51]. In any case, the use of *E. tapos* in yoghurt (HYT5, HYT50, and HYT500) has been shown to reduce maternal obesity-related stress hormones and lower anxiety levels in obese dams. A recent study shows that *E. tapos* can lower cortisol levels. In these, the adrenal cortex stimulates the ACTH to release cortisol, a form of glucocorticoid that is also a primary stress hormone. Cortisol may cause white adipose tissue redistribution in the abdominal region and often stimulates hunger, thereupon promoting weight gain [52]. Nonetheless, the hypothalamus is involved in the regulation of the hypothalamic-pituitary-adrenal (HPA) axis, which is a major stress response system in the body. Activation of the HPA axis can lead to the release of stress hormones such as cortisol, which can have profound effects on brain function and behavior. In the context of anxiety, high levels of cortisol have been linked to increased anxiety symptoms [53]. Along with this, the treatment of *E. tapos* in yoghurt (HYT5, HYT50, and HYT500) alleviates hippocampal-dependent spatial memory in obese dams in this experimental study. The HFD-induced obese dams (HS and HY groups) spend a significantly lower amount of time exploring novel objects and places. This indicates maternal obesity is closely associated with hippocampal dysfunction, which often manifests as memory impairment [54]. The lower levels of GSH and FRAP in the hypothalamus and serum that were observed in the dams of the HS group in this study may be one cause of memory impairment in untreated obese dams. This is consistent with earlier observations made by Sekler et al. (2008) and Goutzourelas et al. (2018): a loss in cellular antioxidants such as FRAP [55] and GSH [56] is a sign of oxidative damage, which frequently shows up as cognitive decline. When an individual’s lipid profile is dysregulated [55] or obese [56], these changes frequently occur. However, the high phenolic contents, such as flavonoids, a strong antioxidant present in *E. tapos* yoghurt [15], were able to reverse the FRAP and GSH deterioration in HFD-induced obese dams, as seen in HYT5, HYT50, and HYT500. This proves the hypothesis that this is achieved as *E. tapos* yoghurt alleviates hippocampal-dependent cognitive deficits and anxiety in HFD-induced obese dams.

## 5. Conclusions

In this study, *E. tapos* in yoghurt supplementation (HYT500) during the gestation period was found to be effective in reducing hyperlipidemia, BMI, abdominal circumference, hyperglycemia, and oxidative stress in the hypothalamus. However, the anxiolytic effect of HYT500 could have been confounded by non-specific effects on movement. Nevertheless, the reduced oxidative stress in the hypothalamus could have provided some sort of neuroprotection, as seen in the NORT and place recognition results. Similarly, HYT50 reduced hyperlipidemia, hyperglycemia, and obesity. It was also found to be anxiolytic and improve NORT and place recognition. However, HYT50 had no significant effect on any of the oxidative parameters tested in the hypothalamus, although it effectively reduced peripheral oxidative stress markers. In totality, the novel formulation of our *E. tapos* in yoghurt at a dose of 500 mg/kg/day has been proven to exhibit anti-obesity effects in maternal obesity, relieve anxiety, and boost hippocampal-dependent memory. However, it is highly recommended to explore the effect of *E. tapos* in yoghurt on different brain regions and different behavioral studies.

## Figures and Tables

**Table 1:** Bioactive compounds of *E. tapos* yoghurt.

No	Molecule	Formula	Class	Molecular Weight (Da)	Observed Molecular Weight (Da)	Observed *m*/*z*	Mass Error (ppm)	Observed RT (min)
1	Meso-inositol	C_6_H_12_O_6_	Carbocyclic sugar Monosaccharide	180.06339	180.0627	179.0555	−3.6	2.55
2	Scropolioside A	C_35_H_44_O_18_	Monoterpenoids Iridoid glycosides	752.25276	752.251	751.2437	−2.4	8.84
3	5′-Methoxy-bilobetin	C_32_H_22_O_11_	Bioflavonoids and polyflavonoids	582.11621	582.1163	581.109	0.2	0.97
4	Galactose	C_6_H_12_O_6_	Monosaccharide sugar	180.06339	180.0629	179.0556	−2.9	0.7
5	Rehmannioside A	C_21_H_32_O_15_	Carotenoid glycoside	524.17412	524.174	523.1668	−0.2	6.02
6	Ephedradine B	C_29_H_38_N_4_O_5_	Spermine alkaloid	522.28422	522.2844	521.2771	0.3	9.29
7	Indigoticoside A	C_26_H_34_O_11_	Phenylpropanoid	522.21011	522.2104	521.2031	0.5	9.79
8	Mannotriose	C_18_H_32_O_16_	Oligosaccharides	504.16903	504.1696	503.1624	1.2	0.66
9	Bruceine B	C_23_H_28_O_11_	Triterpenoid	480.16316	480.165	479.1578	3.9	15.25
10	Forsythoside D	C_20_H_30_O_13_	Phenylethanoid glycosides	478.16864	478.1685	477.1612	−0.3	6.02
11	2,3,5,4′-Tetrahydroxystilbene- 2-O-(6″-O-acetyl)-β-D-glucopyranoside	C_22_H_24_O_10_	Glycoside (Flavonoids)	448.13695	448.1364	447.1292	−1.2	2.73
12	Asperulosidic acid	C_18_H_24_O_12_	Glycoside Iridoid monoterpenoid	432.12678	432.1269	431.1196	0.3	4.2
13	Asperuloside	C_18_H_22_O_11_	Iridoid monoterpenoid glycoside	414.11621	414.1166	413.1093	1	10.12
14	Apocynoside Ⅰ	C_19_H_30_O_8_	Ionone glucoside	386.19407	386.1941	385.1868	0	6.74
15	Isomaltose	C_12_H_22_O_11_	Disaccharide	342.11621	342.1161	341.1089	−0.2	0.69
16	Flazin	C_17_H_12_N_2_O_4_	Harmala alkaloid	308.07971	308.0793	307.072	−1.4	12.84
17	Astragaline E	C_14_H_16_N_2_O_5_	kaempferol-3-O-β-d-glucoside Flavonoid	292.10592	292.1059	291.0986	−0.2	4.55
18	Tribulusterine	C_16_H_12_N_2_O_2_	β-carboline alkaloid	264.08988	264.0894	263.0821	−2	12.84
19	Sinapic acid	C_11_H_12_O_5_	Hydroxycinnamic acid Phenylpropanoid	224.06847	224.0679	223.0606	−2.7	5.92
20	Tyrosine	C_9_H_11_NO_3_	Nonessential amino acid	181.07389	181.0735	180.0662	−2.1	1.07

## Data Availability

The corresponding author can provide access to the dataset that was generated and/or analyzed during the present study upon request, subject to reasonable conditions.

## Supplementary Materials

The following supporting information can be downloaded at: https://www.mdpi.com/article/10.3390/nu15102312/s1, Figure S1: Comprehensive peak characterization trace of Meso-inositol present in *E. tapos* yoghurt from TWIMS-QTOFMS analysis.; Figure S2: Comprehensive peak characterization trace of Scropolioside A present in *E. tapos* yoghurt from TWIMS-QTOFMS analysis; Figure S3: Comprehensive peak characterization trace of 5′-Methoxy-bilobetin present in *E. tapos* yoghurt from TWIMS-QTOFMS analysis; Figure S4: Comprehensive peak characterization trace of Galactose present in *E. tapos* yoghurt from TWIMS-QTOFMS analysis; Figure S5: Comprehensive peak characterization trace of Rehmannioside A present in *E. tapos* yoghurt from TWIMS-QTOFMS analysis; Figure S6: Comprehensive peak characterization trace of Ephedradine B present in *E. tapos* yoghurt from TWIMS-QTOFMS analysis; Figure S7: Comprehensive peak characterization trace of Indigoticoside A present in *E. tapos* yoghurt from TWIMS-QTOFMS analysis; Figure S8: Comprehensive peak characterization trace of Mannotriose present in *E. tapos* yoghurt from TWIMS-QTOFMS analysis; Figure S9: Comprehensive peak characterization trace of Bruceine B present in *E. tapos* yoghurt from TWIMS-QTOFMS analysis; Figure S10: Comprehensive peak characterization trace of Forsythoside D present in *E. tapos* yoghurt from TWIMS-QTOFMS analysis; Figure S11: Comprehensive peak characterization trace of 2,3,5,4′-Tetrahydroxystilbene-2-O-(6″-O-acetyl)-β-D-glucopyranoside present in *E. tapos* yoghurt from TWIMS-QTOFMS analysis; Figure S12: Comprehensive peak characterization trace of Asperulosidic acid present in *E. tapos* yoghurt from TWIMS-QTOFMS analysis; Figure S13: Comprehensive peak characterization trace of Asperuloside present in *E. tapos* yoghurt from TWIMS-QTOFMS analysis.; Figure S14: Comprehensive peak characterization trace of Apocynoside Ⅰ present in *E. tapos* yoghurt from TWIMS-QTOFMS analysis; Figure S15: Comprehensive peak characterization trace of Isomaltose present in *E. tapos* yoghurt from TWIMS-QTOFMS analysis.; Figure S16: Comprehensive peak characterization trace of Flazin present in *E. tapos* yoghurt from TWIMS-QTOFMS analysis; Figure S17: Comprehensive peak characterization trace of Astragaline E present in *E. tapos* yoghurt from TWIMS-QTOFMS analysis; Figure S18: Comprehensive peak characterization trace of Tribulusterine present in *E. tapos* yoghurt from TWIMS-QTOFMS analysis; Figure S19: Comprehensive peak characterization trace of Sinapic acid present in *E. tapos* yoghurt from TWIMS-QTOFMS analysis; Figure S20: Comprehensive peak characterization trace of Tyrosine present in *E. tapos* yoghurt from TWIMS-QTOFMS analysis.
