# Supplementary material for: Elateriospermum tapos Yoghurt as a Therapeutic Intervention for Obesity-Associated Cognitive Impairments and Anxiety-like Behaviour in a High Fat Diet Maternal Obese Rat Model
Source: Nutrients. 2023 May 15;15(10):2312. doi: 10.3390/nu15102312 (PMC10223143; doi:10.3390/nu15102312)
Supplement: Supplementary file 1 [file nutrients-15-02312-s001.zip › nutrients-2379943-supplementary.pdf]

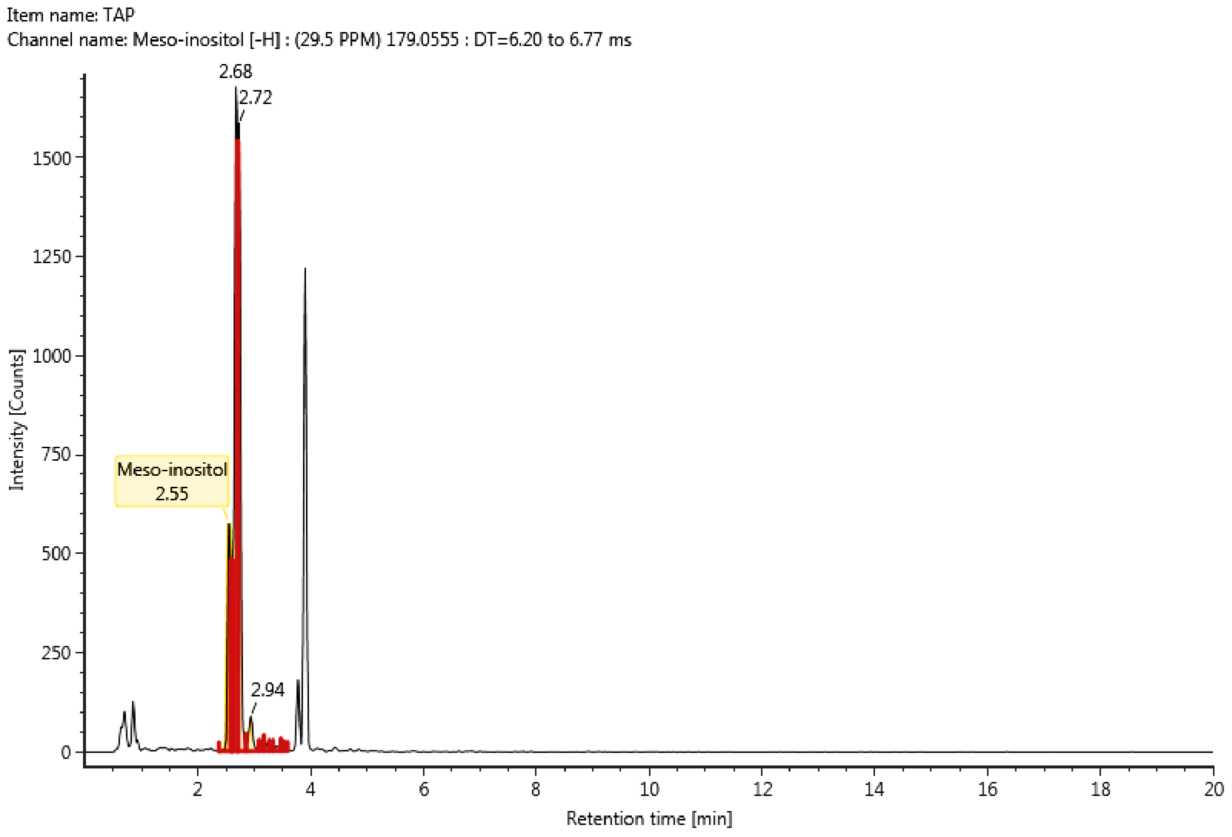

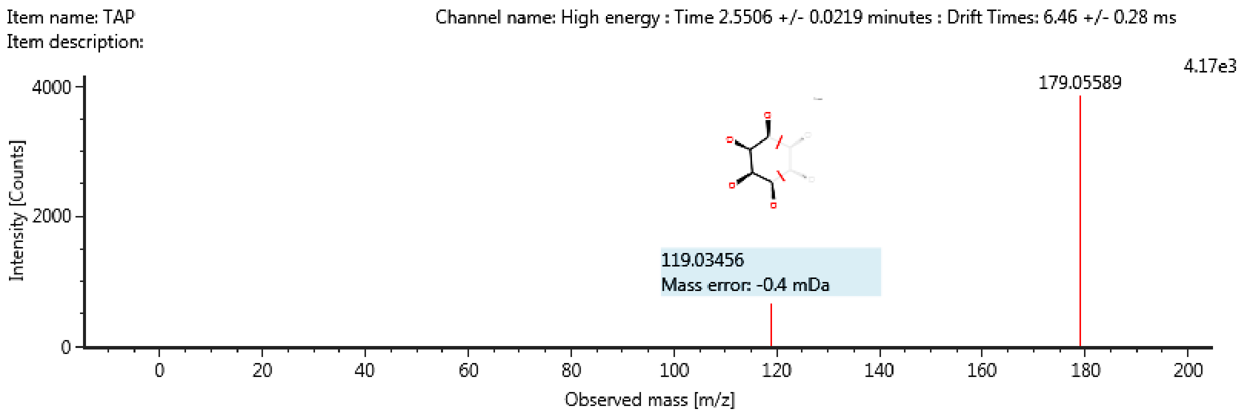

Supplementary figure S1. Comprehensive peak characterization trace of Meso-inositol present in *E. tapos* yoghurt from TWIMS-QTOFMS analysis.

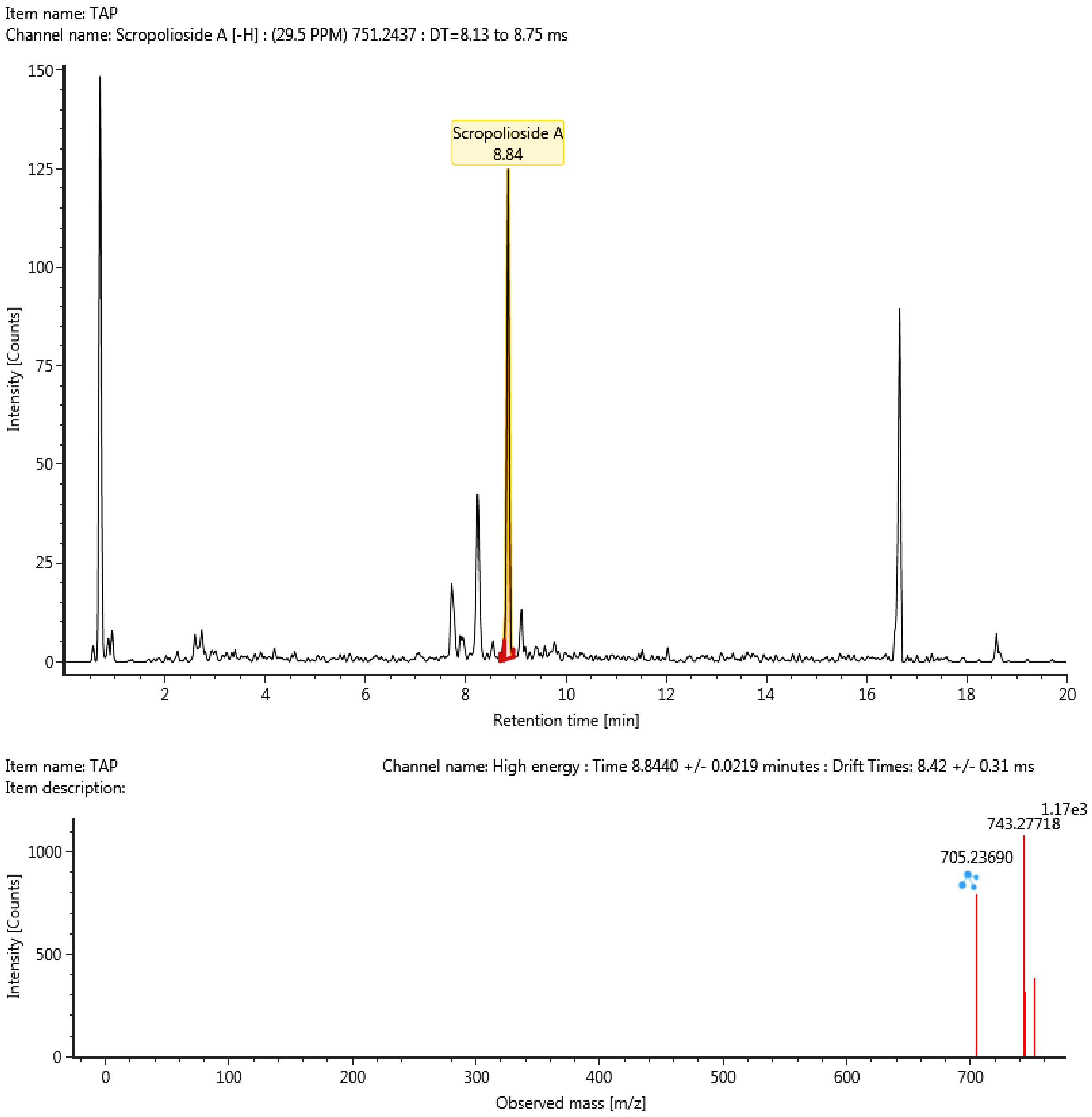

Supplementary figure S2. Comprehensive peak characterization trace of Scropolioside A present in *E. tapos* yoghurt from TWIMS-QTOFMS analysis.

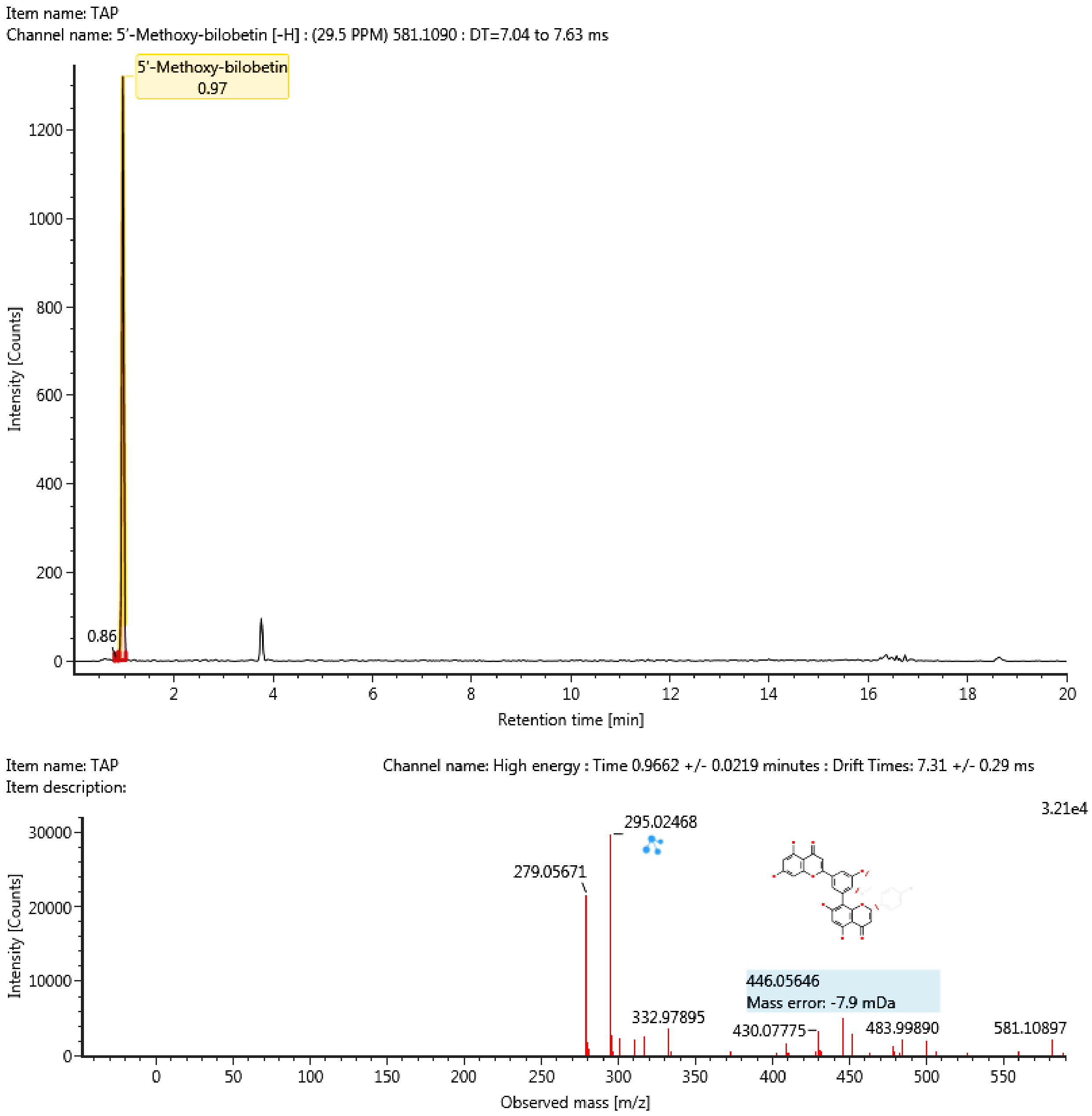

Supplementary figure S3. Comprehensive peak characterization trace of 5′-Methoxy-bilobetin present in *E. tapos* yoghurt from TWIMS-QTOFMS analysis.

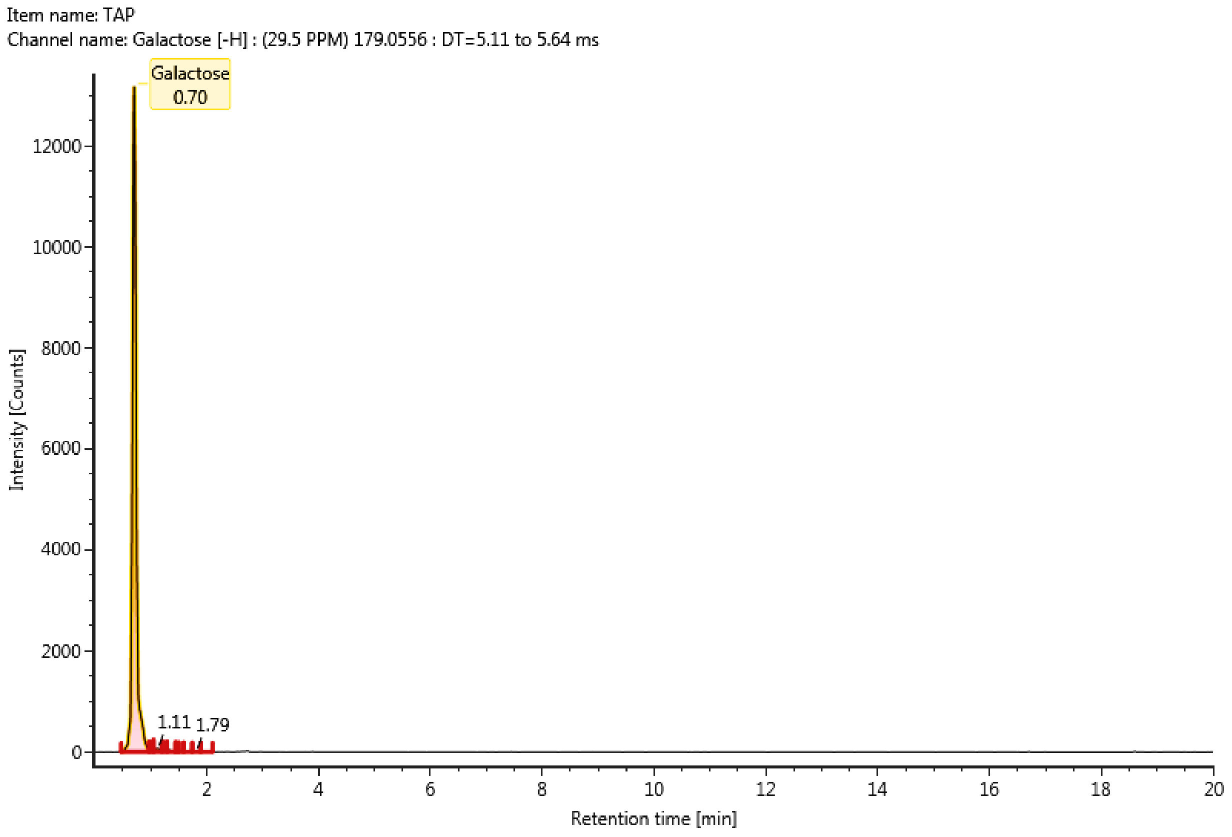

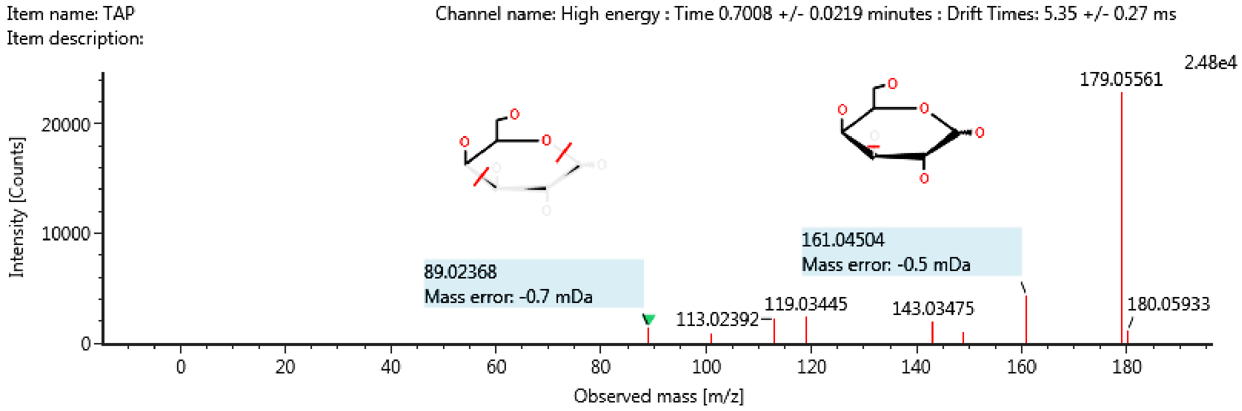

Supplementary figure S4. Comprehensive peak characterization trace of Galactose present in *E. tapos* yoghurt from TWIMS-QTOFMS analysis.

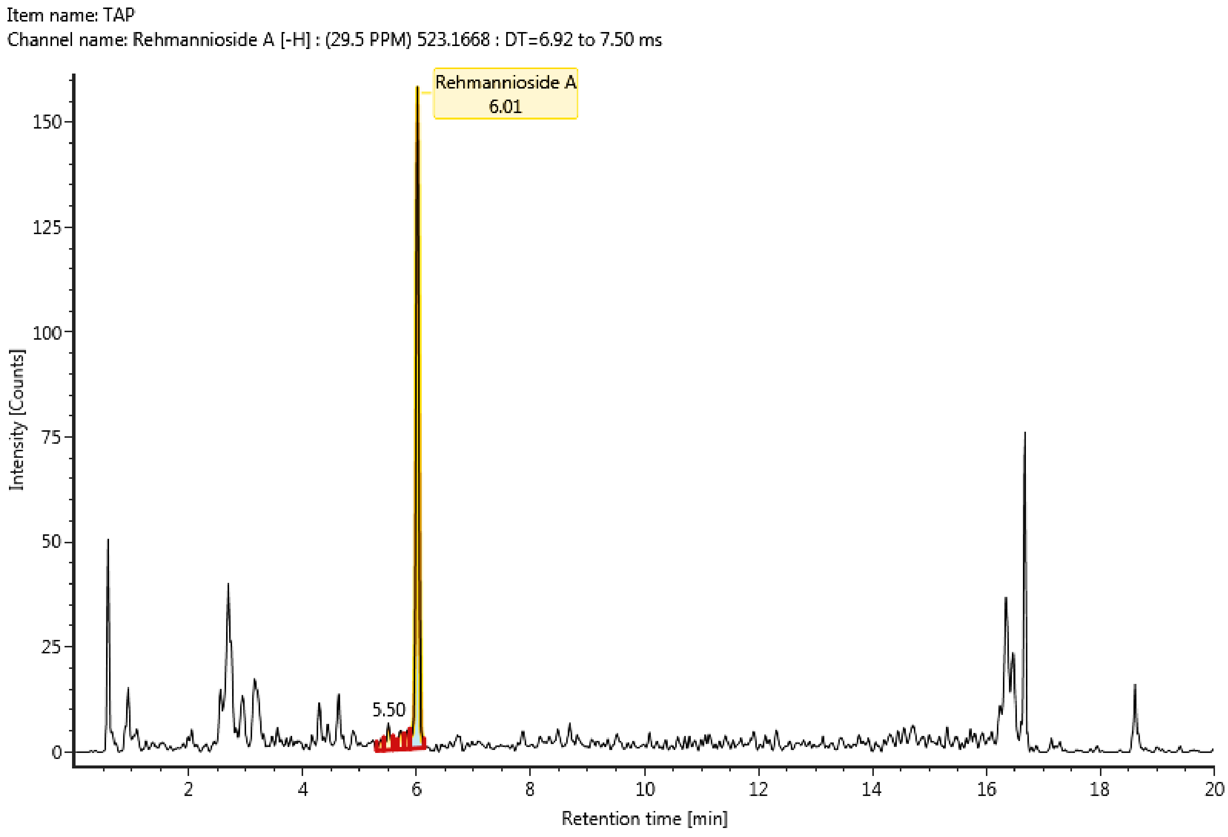

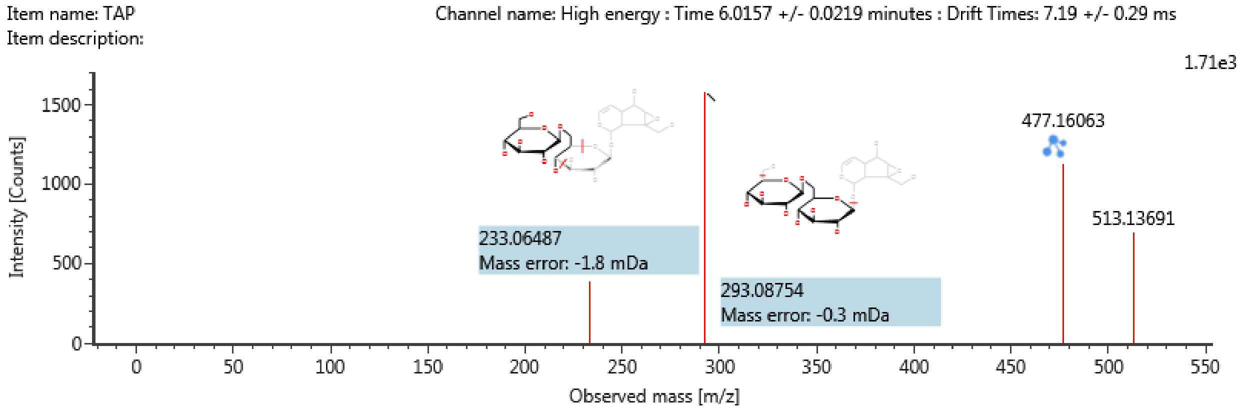

Supplementary figure S5. Comprehensive peak characterization trace of Rehmannioside A present in *E. tapos* yoghurt from TWIMS-QTOFMS analysis.

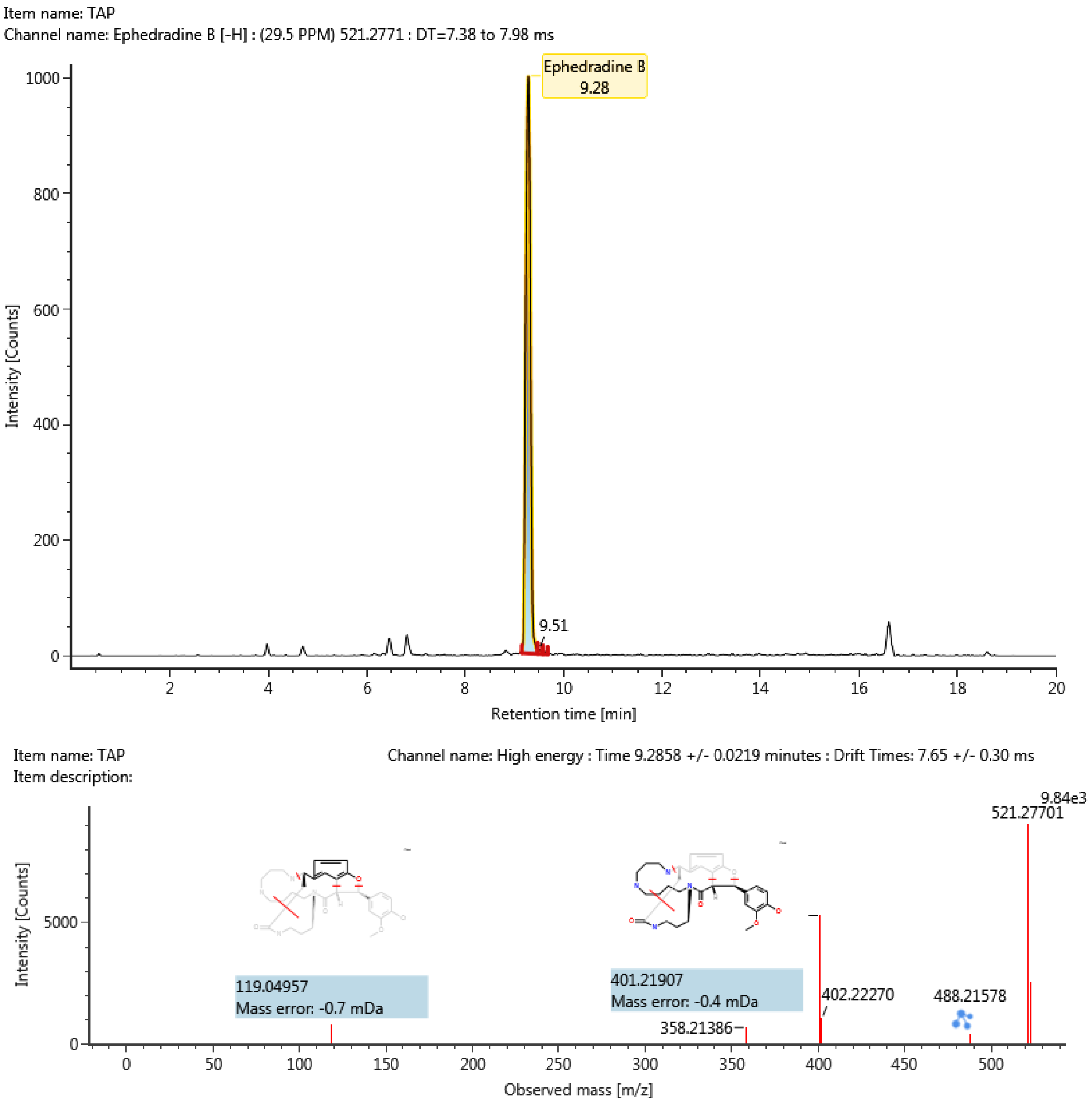

Supplementary figure S6. Comprehensive peak characterization trace of Ephedradine B present in *E. tapos* yoghurt from TWIMS-QTOFMS analysis.

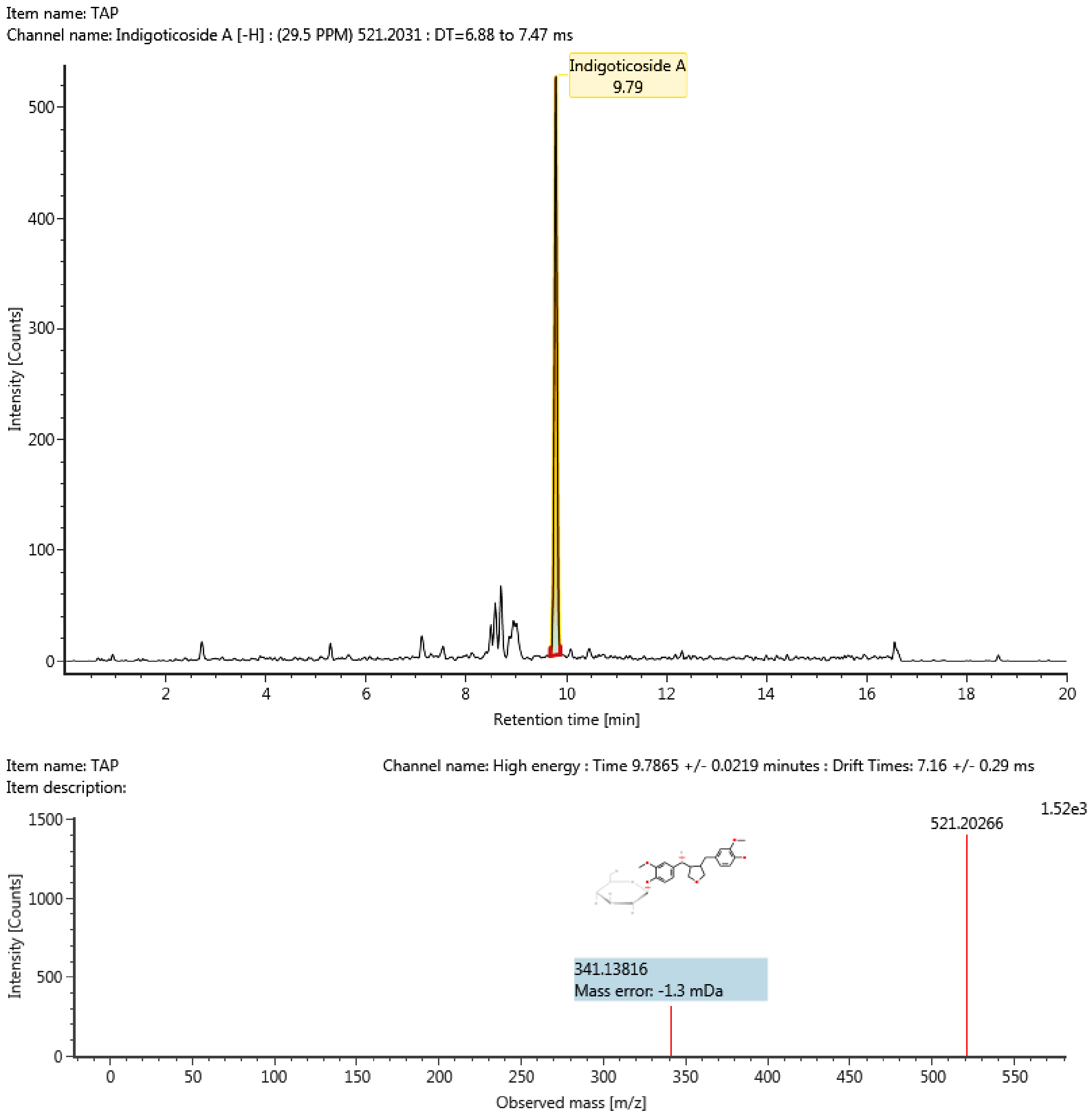

Supplementary figure S7. Comprehensive peak characterization trace of Indigoticoside A present in *E. tapos* yoghurt from TWIMS-QTOFMS analysis.

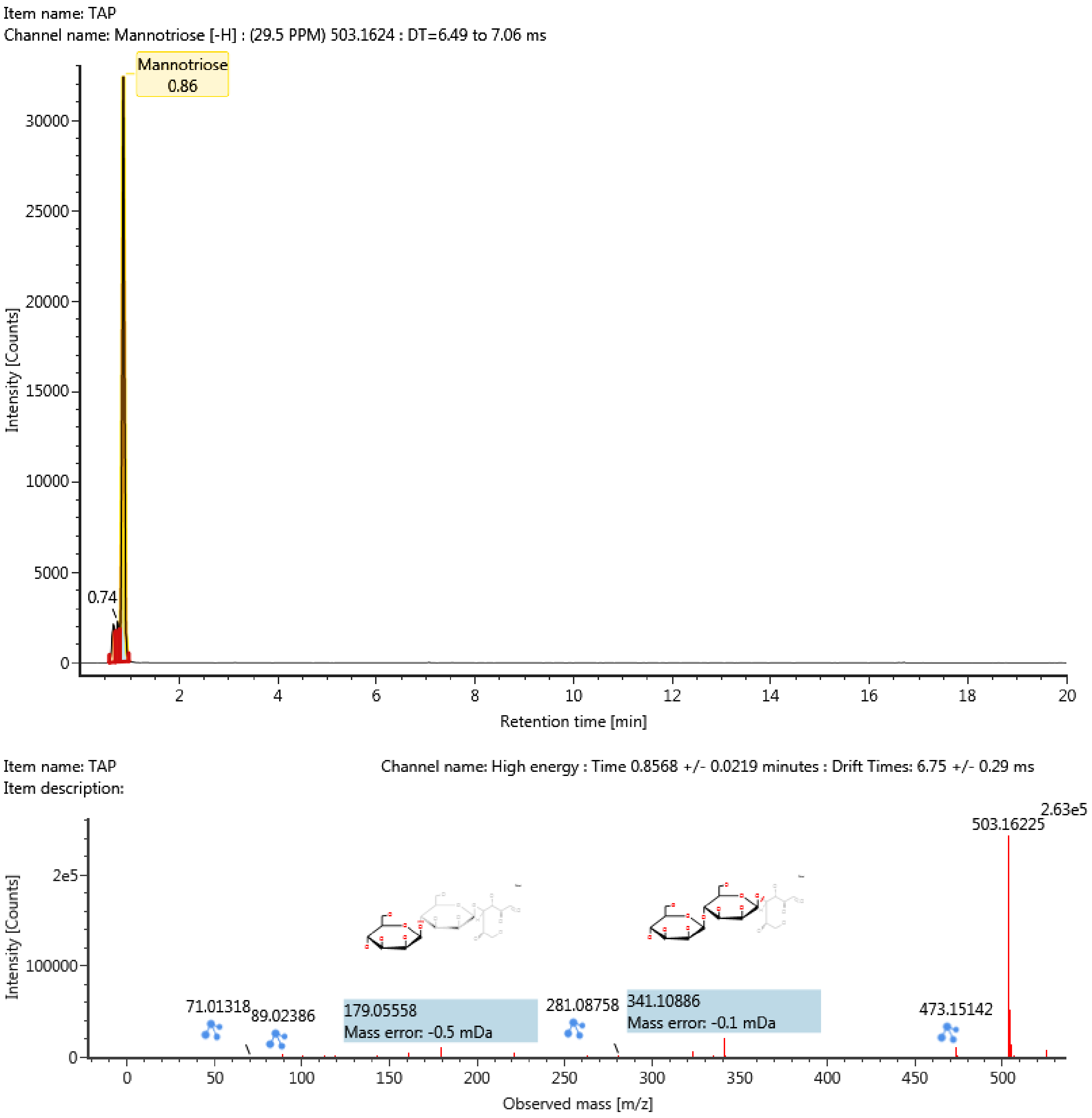

Supplementary figure S8. Comprehensive peak characterization trace of Mannotriose present in *E. tapos* yoghurt from TWIMS-QTOFMS analysis.

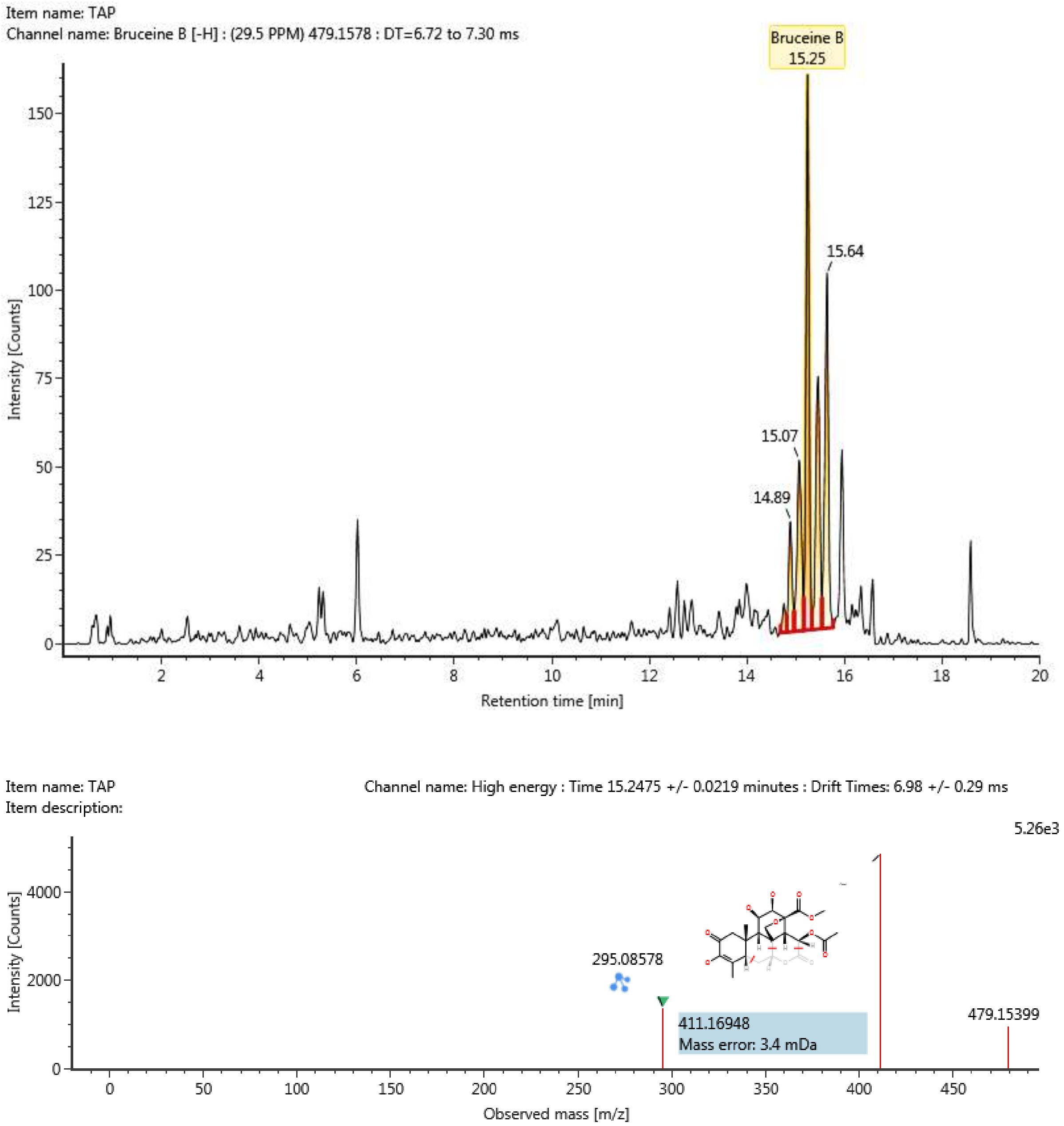

Supplementary figure S9. Comprehensive peak characterization trace of Bruceine B present in *E. tapos* yoghurt from TWIMS-QTOFMS analysis.

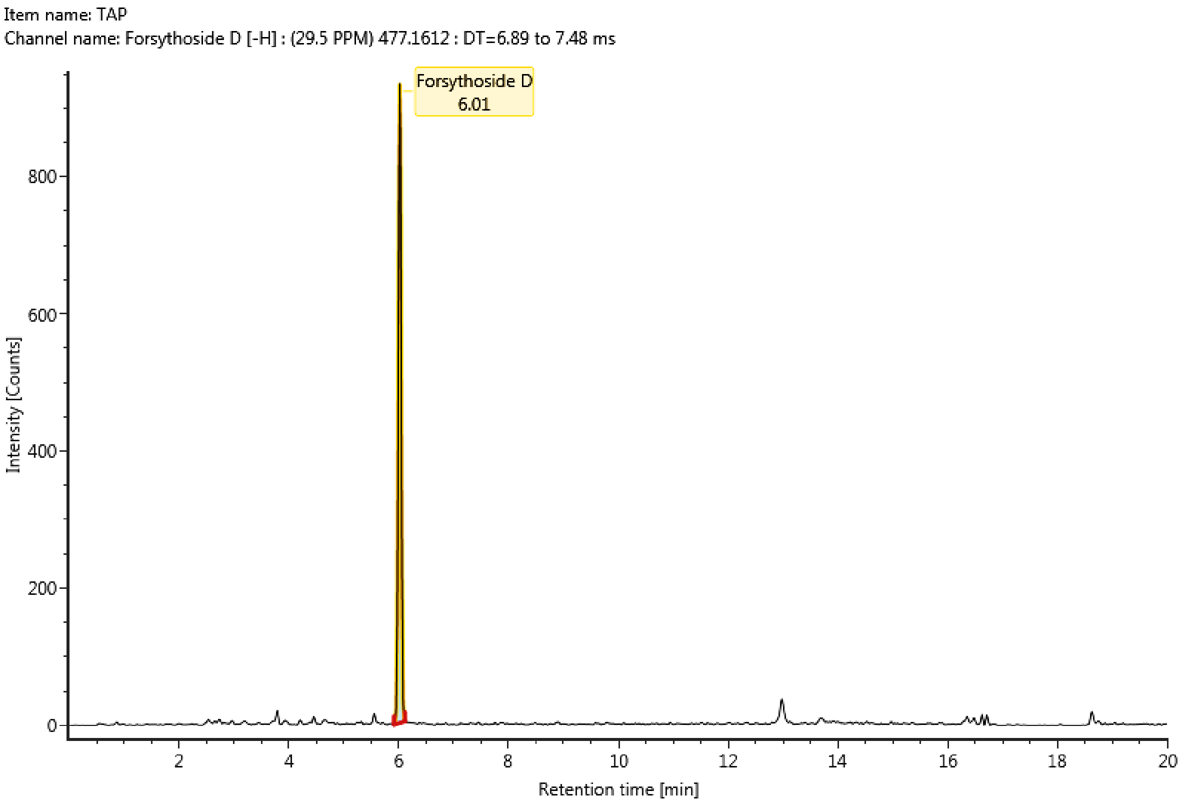

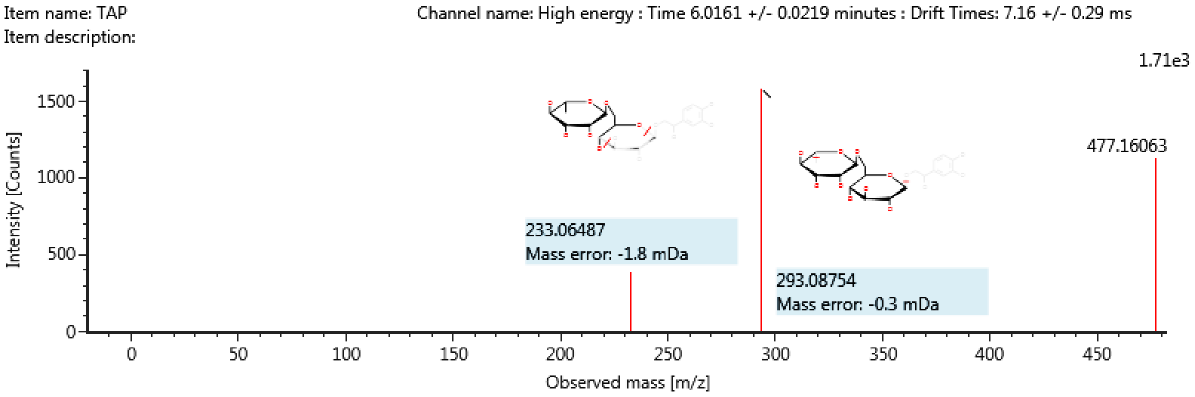

Supplementary figure S10. Comprehensive peak characterization trace of Forsythoside D present in *E. tapos* yoghurt from TWIMS-QTOFMS analysis.

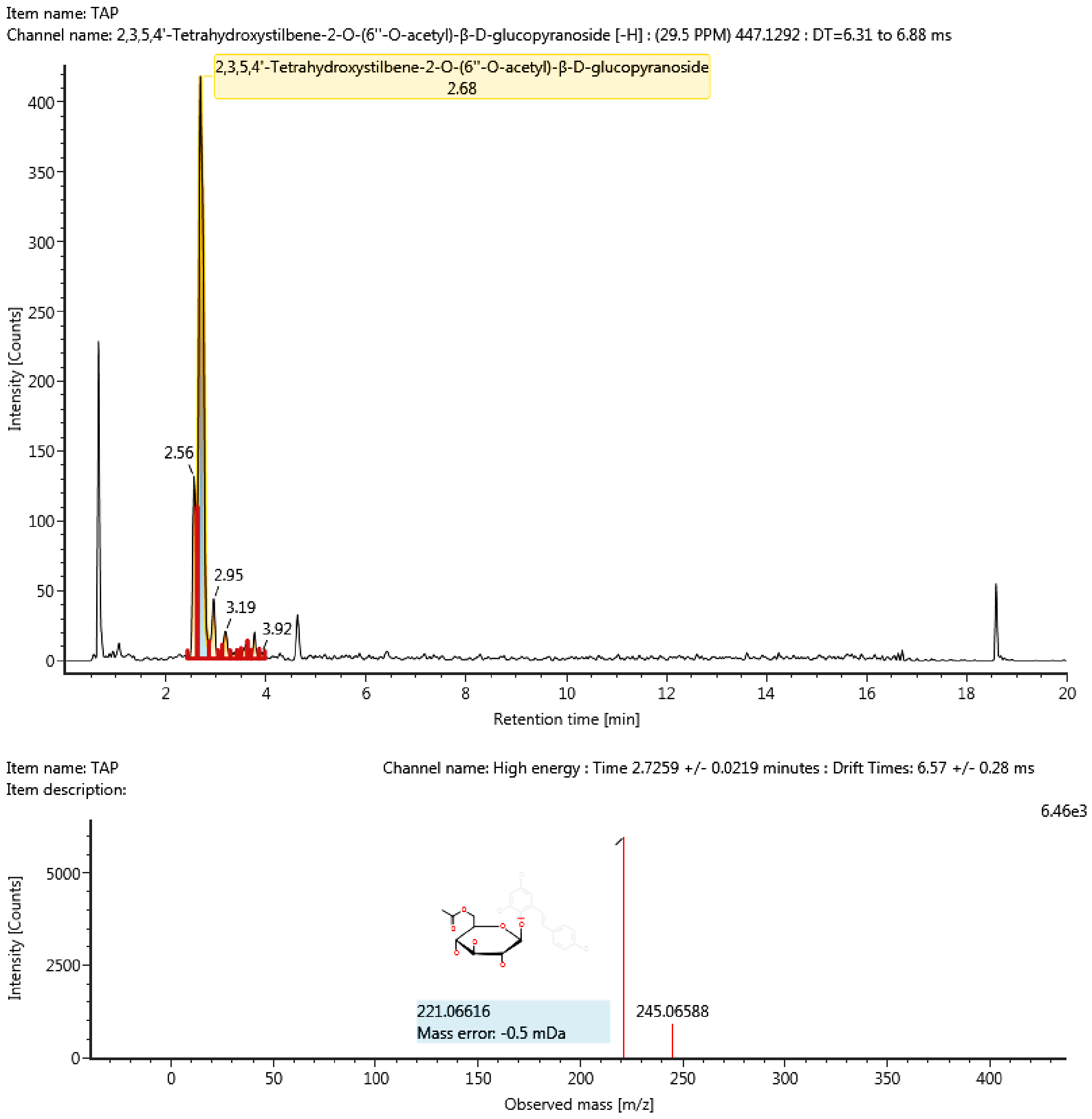

Supplementary figure S11. Comprehensive peak characterization trace of 2,3,5,4'-Tetrahydroxystilbene-2-O-(6''-O-acetyl)-β-D-glucopyranoside present in *E. tapos* yoghurt from TWIMS-QTOFMS analysis.

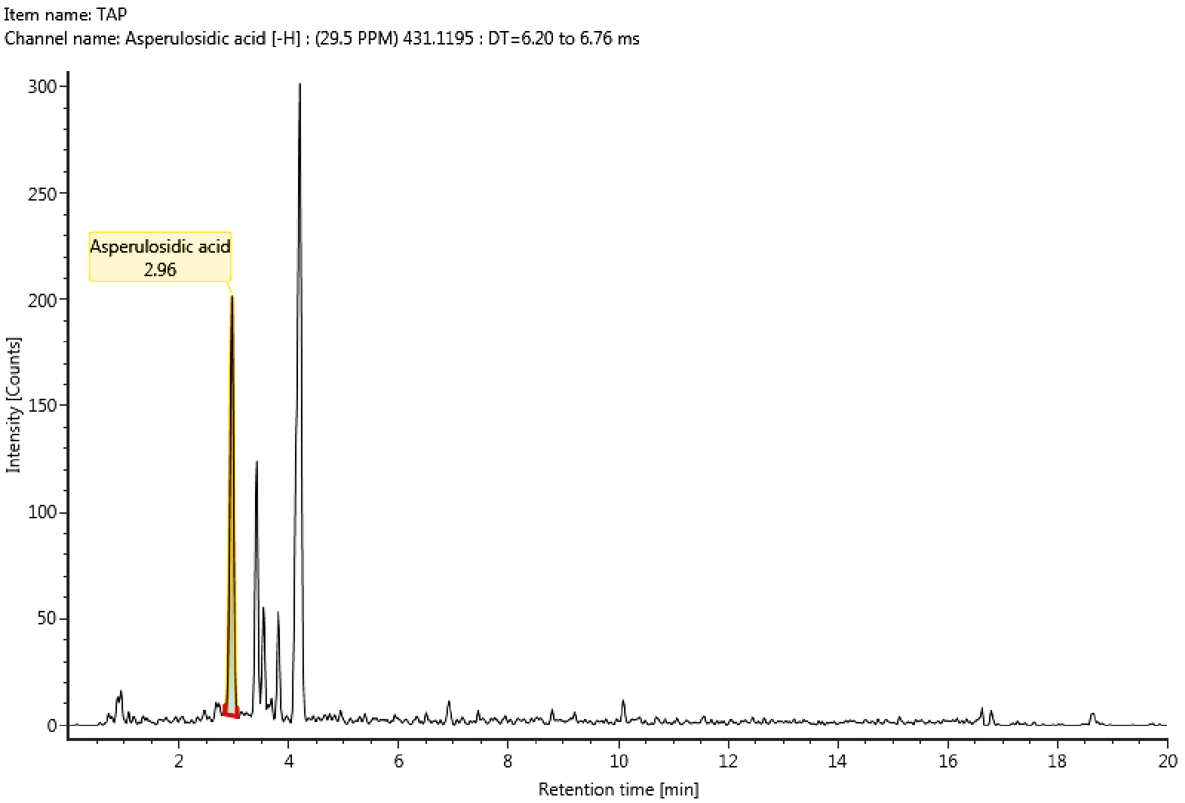

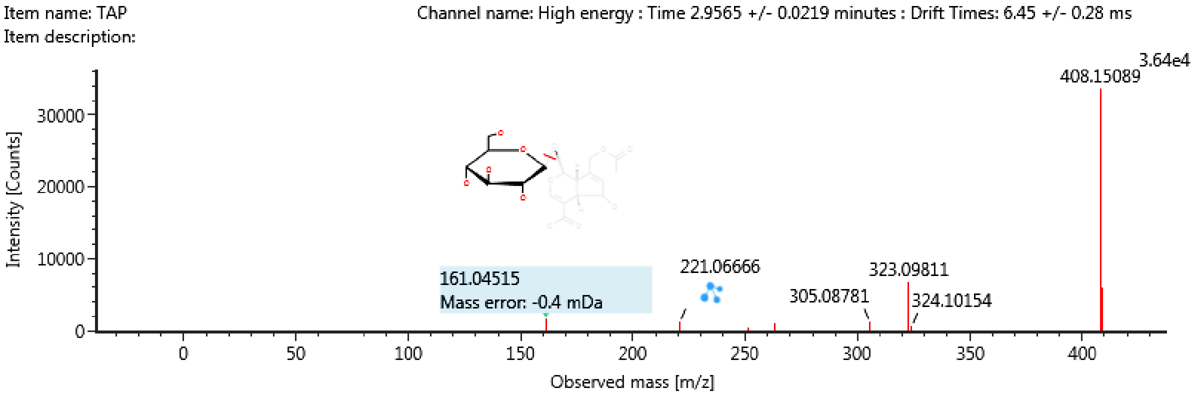

Supplementary figure S12. Comprehensive peak characterization trace of Asperulosidic acid present in *E. tapos* yoghurt from TWIMS-QTOFMS analysis.

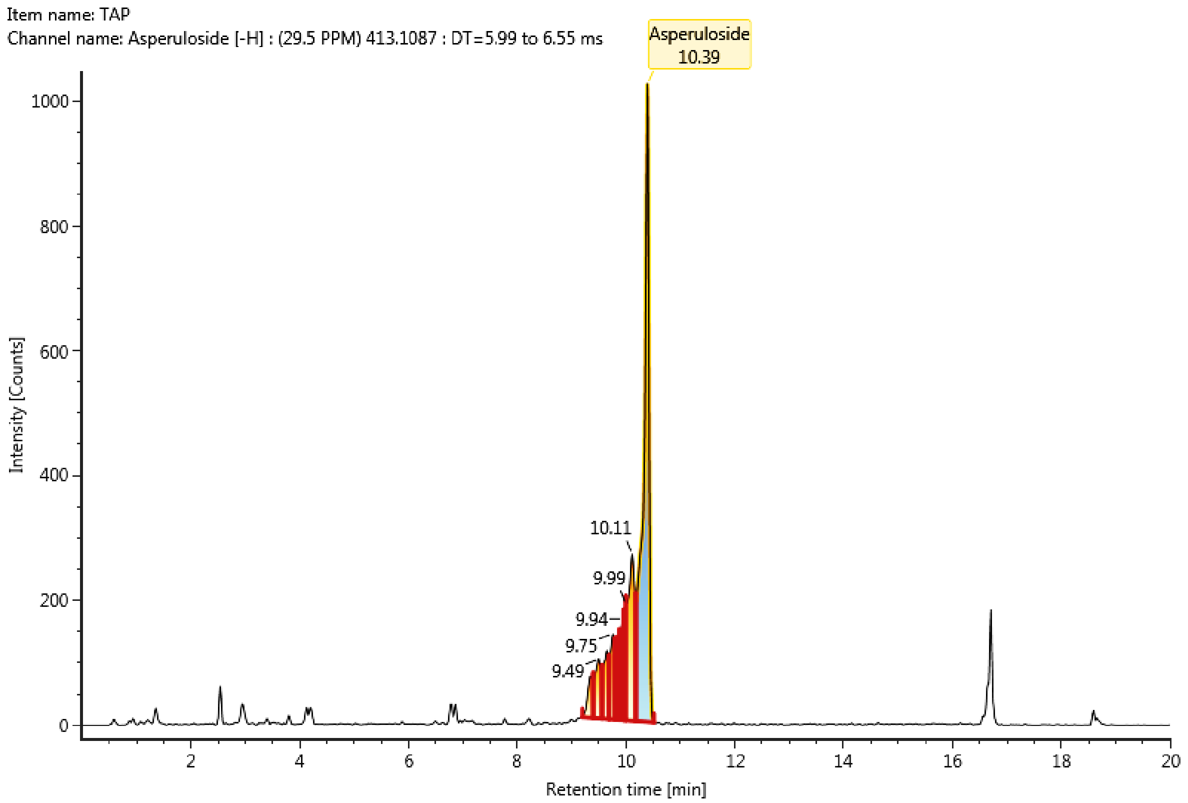

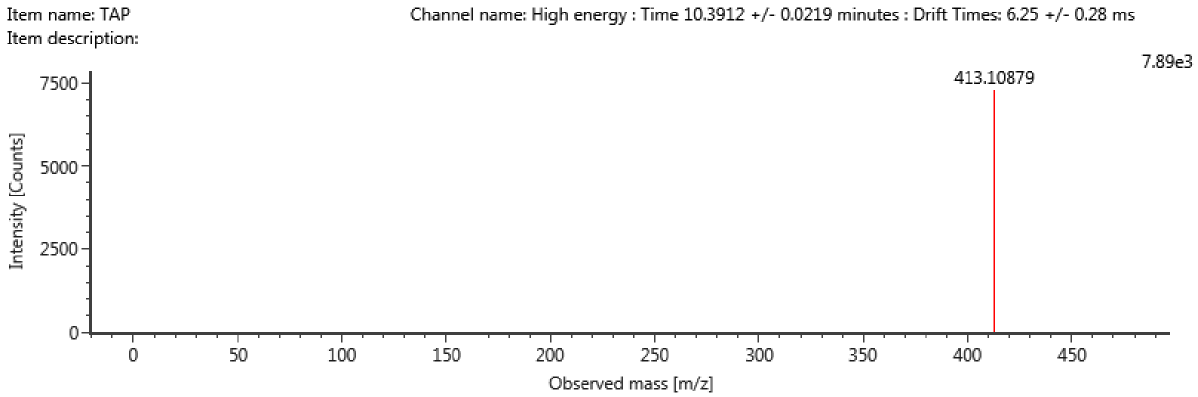

Supplementary figure S13. Comprehensive peak characterization trace of Asperuloside present in *E. tapos* yoghurt from TWIMS-QTOFMS analysis.

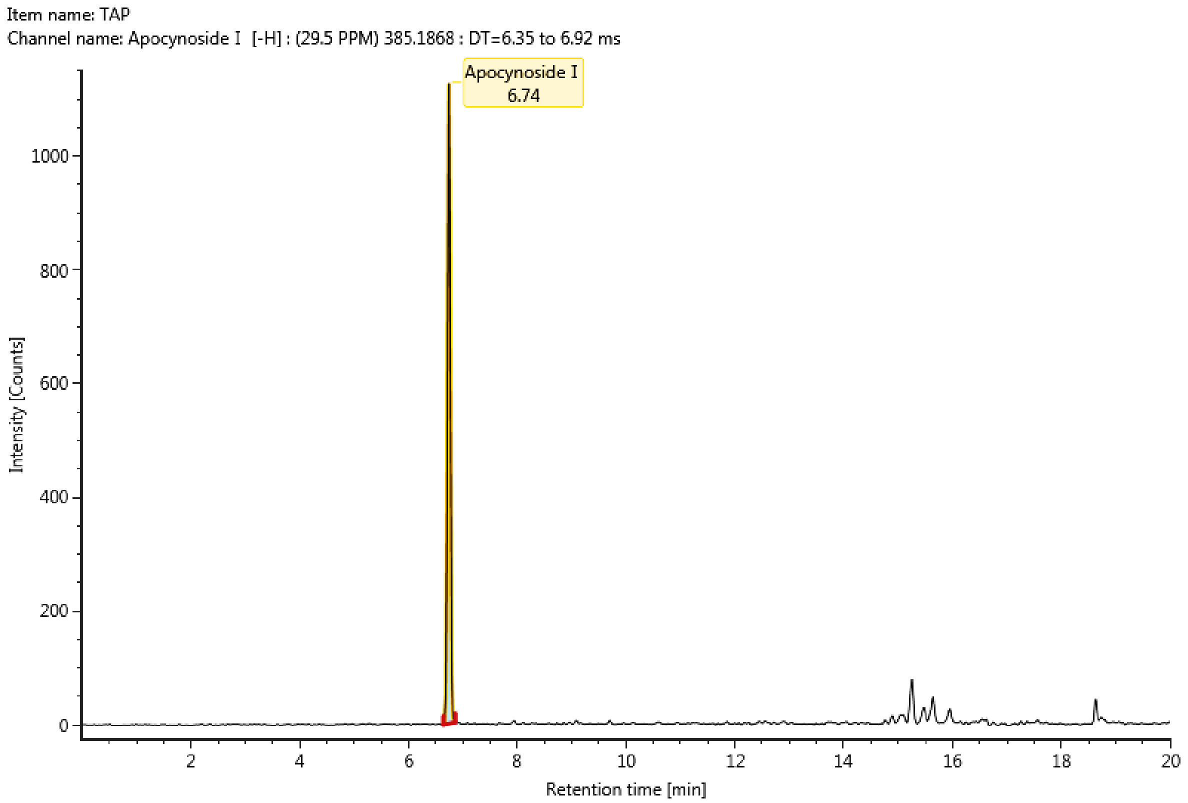

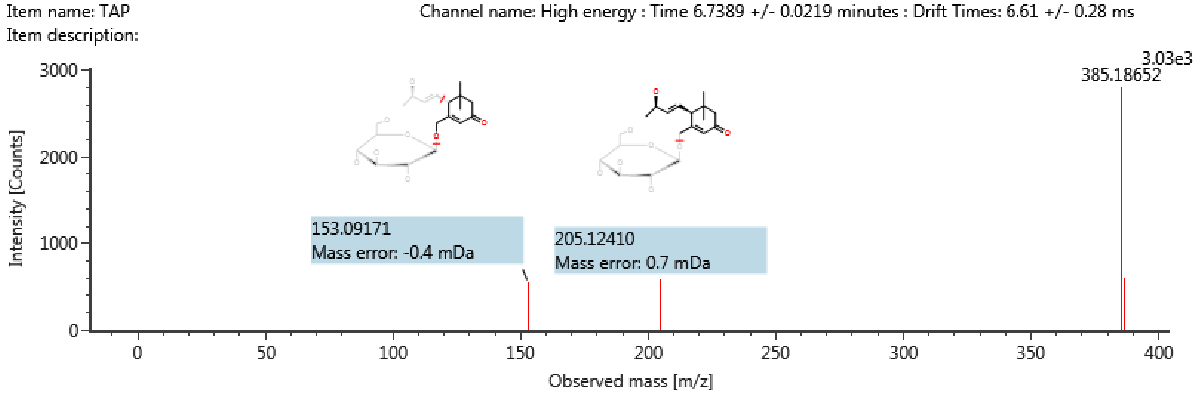

Supplementary figure S14. Comprehensive peak characterization trace o f Apocynoside I present in *E. tapos* yoghurt from TWIMS-QTOFMS analysis.

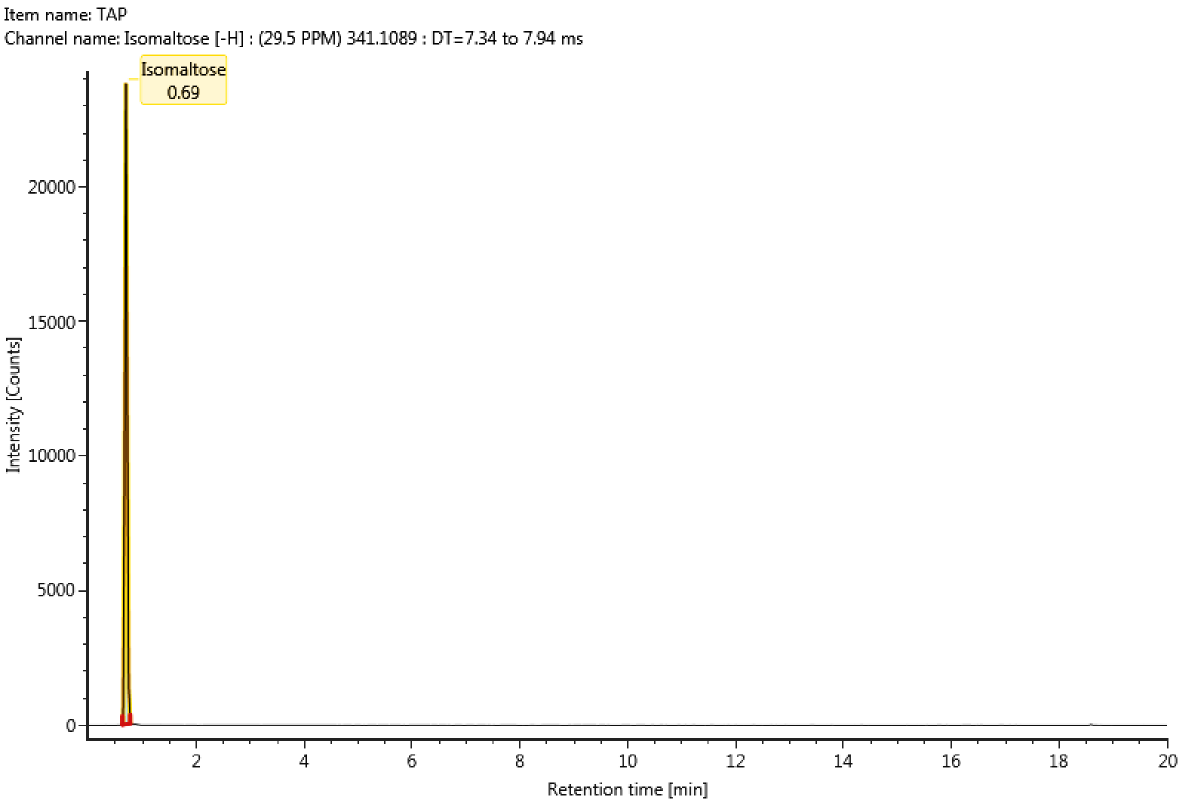

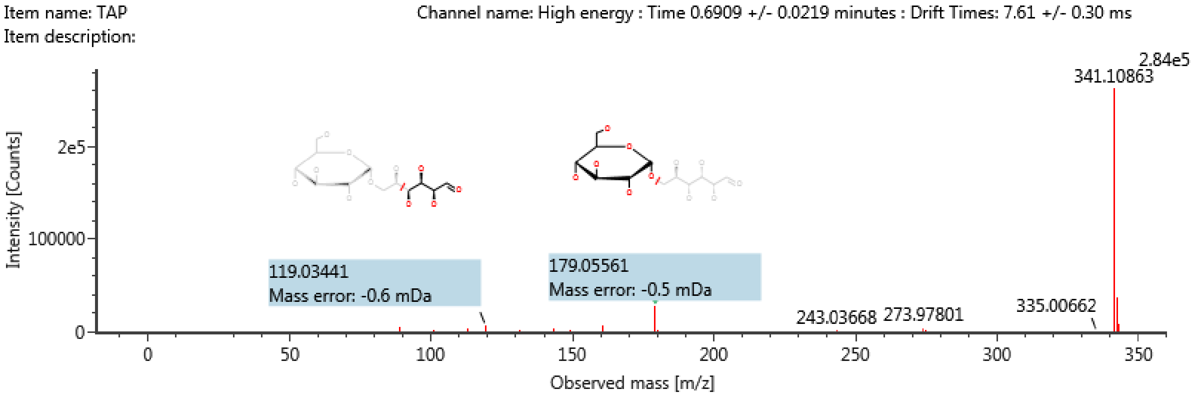

Supplementary figure S15. Comprehensive peak characterization trace of Isomaltose present in *E. tapos* yoghurt from TWIMS-QTOFMS analysis.

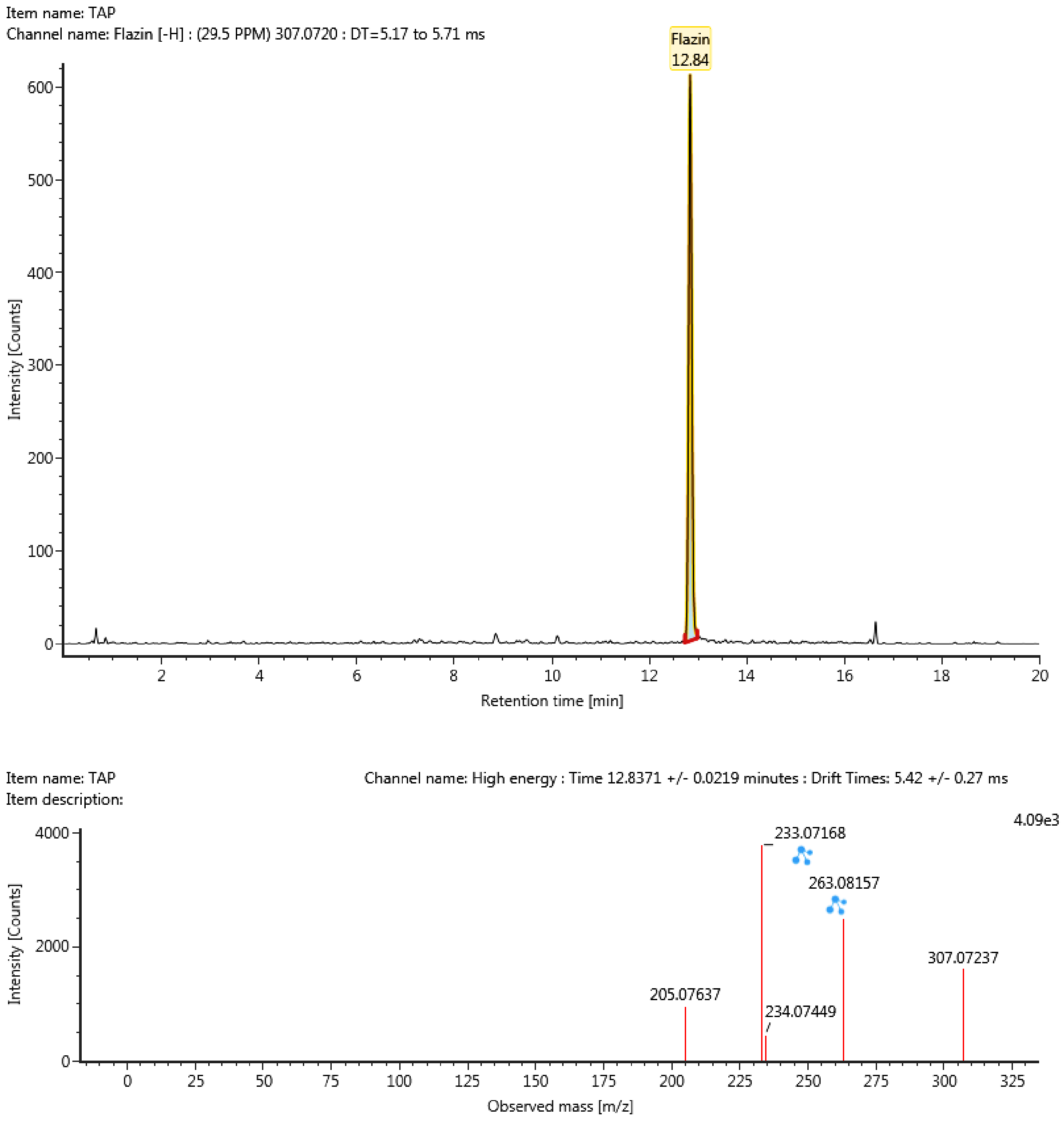

Supplementary figure S16. Comprehensive peak characterization trace of Flazin present in *E. tapos* yoghurt from TWIMS-QTOFMS analysis.

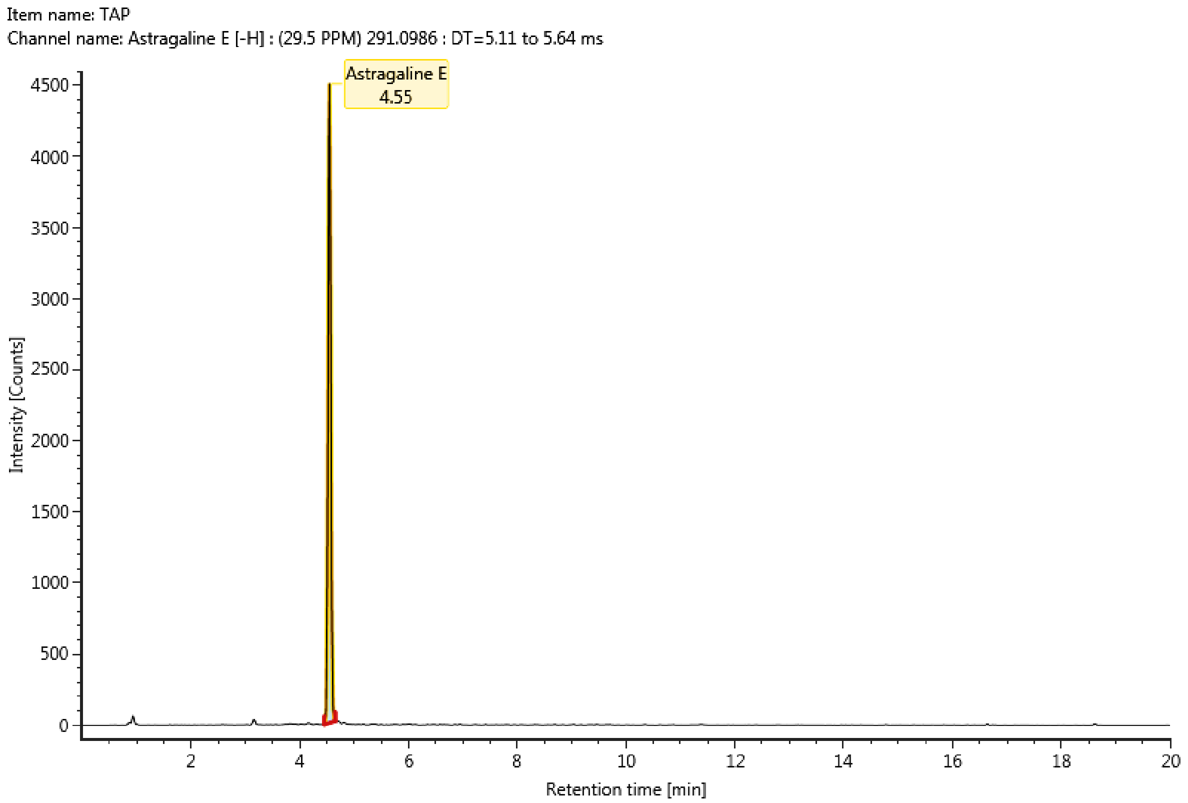

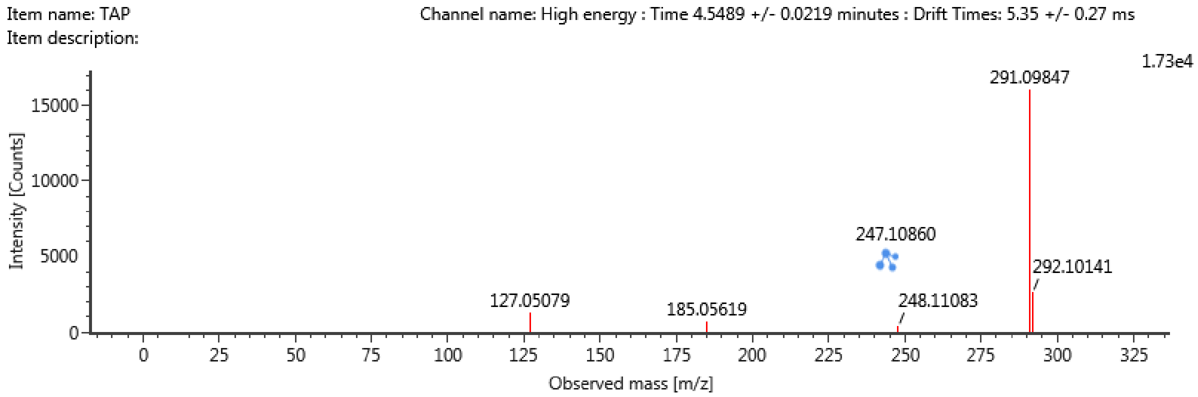

Supplementary figure S17. Comprehensive peak characterization trace of Astragaline E present in *E. tapos* yoghurt from TWIMS-QTOFMS analysis.

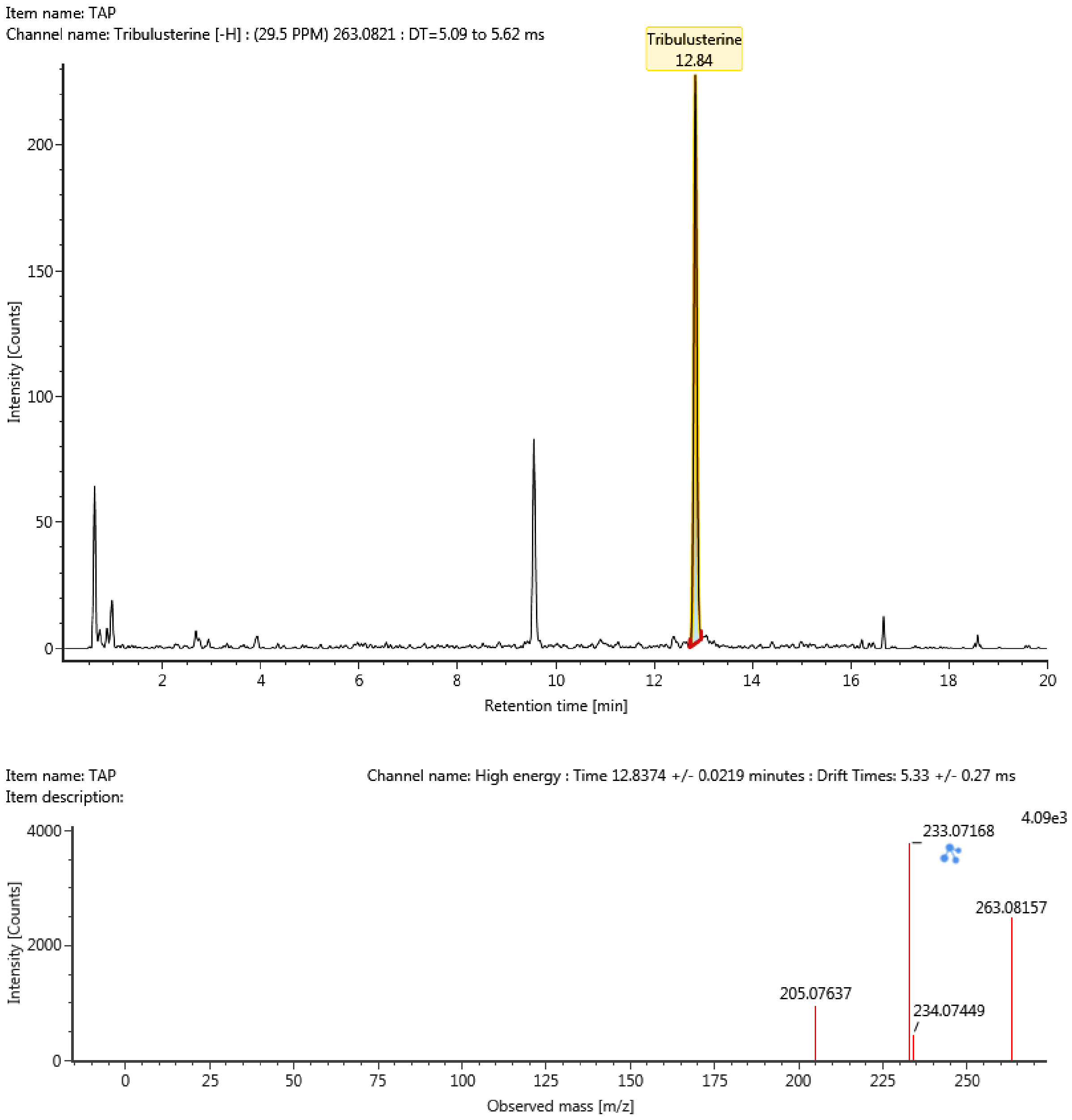

Supplementary figure S18. Comprehensive peak characterization trace of Tribulusterine present in *E. tapos* yoghurt from TWIMS-QTOFMS analysis.

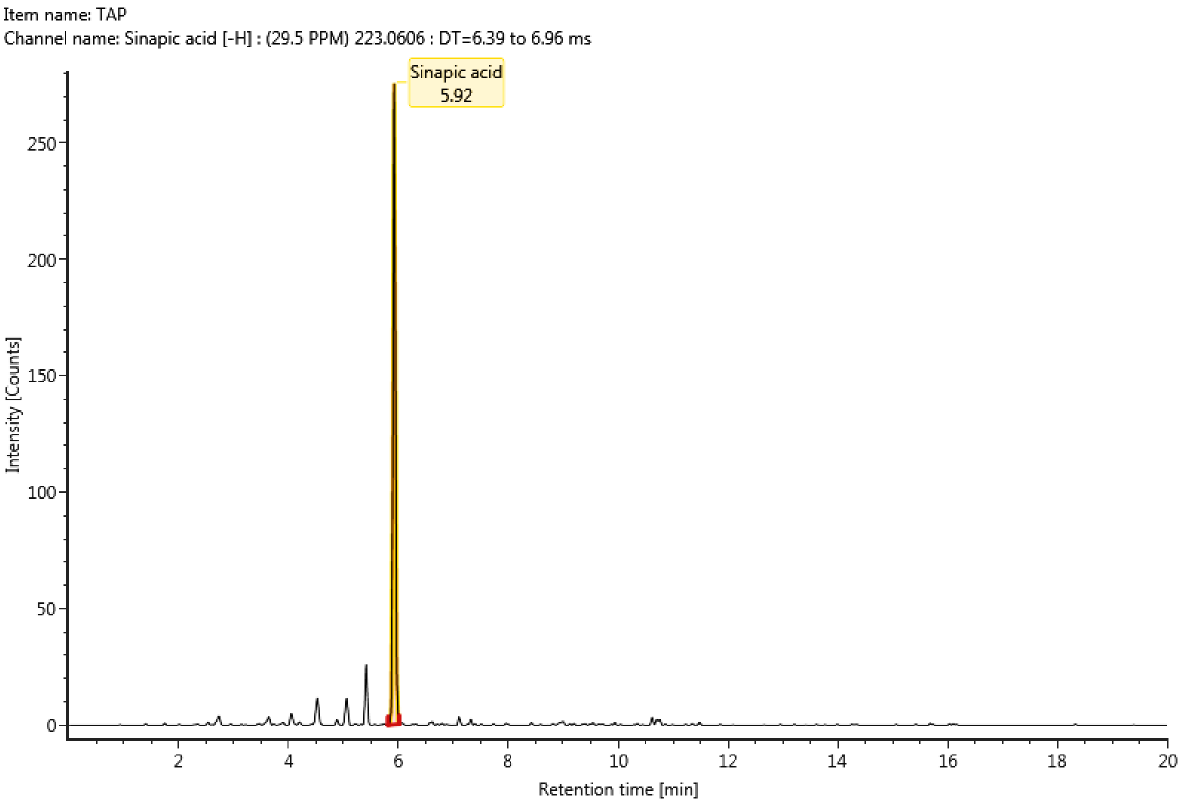

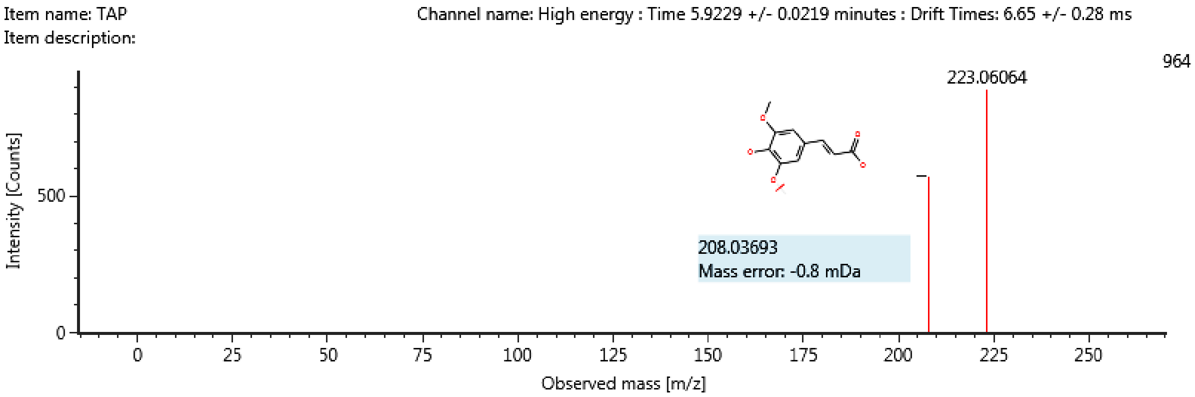

Supplementary figure S19. Comprehensive peak characterization trace of Sinapic acid present in *E. tapos* yoghurt from TWIMS-QTOFMS analysis.

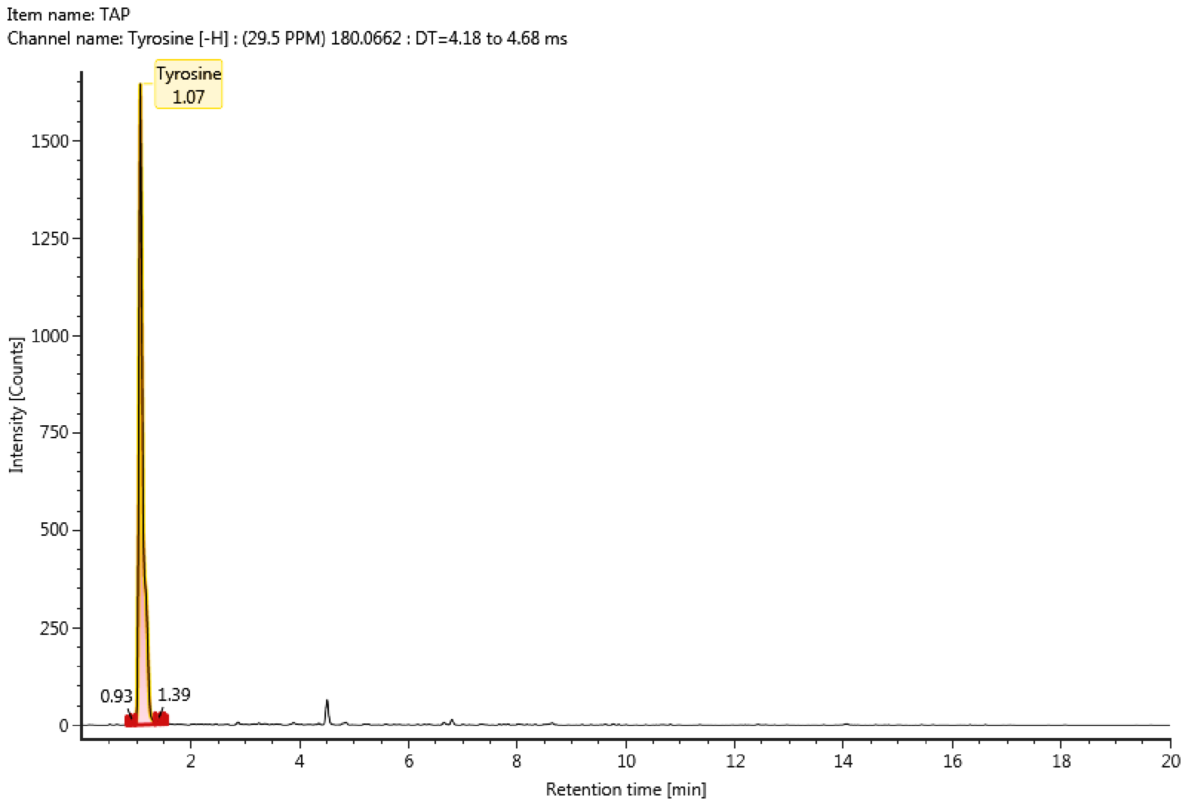

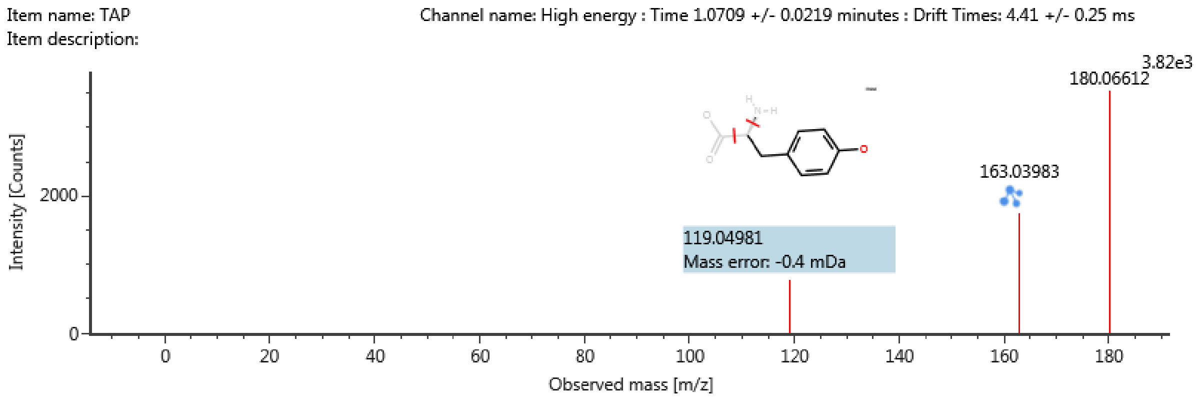

Supplementary figure S20. Comprehensive peak characterization trace of Tyrosine present in *E. tapos* yoghurt from TWIMS-QTOFMS analysis.
